# NMR-Based Metabolite Profiling and the Application of STOCSY toward the Quality and Authentication Assessment of European EVOOs

**DOI:** 10.3390/molecules28041738

**Published:** 2023-02-11

**Authors:** Stavros Beteinakis, Anastasia Papachristodoulou, Peter Kolb, Paul Rösch, Stephan Schwarzinger, Emmanuel Mikros, Maria Halabalaki

**Affiliations:** 1 Division of Pharmacognosy and Natural Products Chemistry, Department of Pharmacy, National and Kapodistrian University of Athens, Panepistimiopolis, Zografou, 15771 Athens, Greece; 2 NBNC—North Bavarian NMR Centre, University of Bayreuth, Universitätsstraße 30, 95447 Bayreuth, Germany; 3 ALNuMed GmbH, Gottfried-Keim-Strasse 60, 95448 Bayreuth, Germany; 4 ForN—Research Unit for Food Quality, University of Bayreuth, Universitätsstraße 30, 95447 Bayreuth, Germany; 5 FLMR—Research Unit for German and European Food Law, University of Bayreuth, Universitätsstraße 30, 95447 Bayreuth, Germany; 6 Division of Pharmaceutical Chemistry, Department of Pharmacy, National and Kapodistrian University of Athens, Panepistimiopolis, Zografou, 15771 Athens, Greece

**Keywords:** *Olea europaea* L., extra virgin olive oil—EVOO, NMR spectroscopy, metabolite profiling, STOCSY, geographical origin, botanical origin, biomarkers, foodomics

## Abstract

Extra virgin olive oil (EVOO) possesses a high-value rank in the food industry, thus making it a common target for adulteration. Hence, several methods have been essentially made available over the years. However, the issue of authentication remains unresolved with national and food safety organizations globally struggling to regulate and control its market. Over the course of this study, the aim was to determine the origin of EVOOs suggesting a high-throughput, state-of-the-art method that could be easily adopted. A rapid, NMR-based untargeted metabolite profiling method was applied and complemented by multivariate analysis (MVA) and statistical total correlation spectroscopy (STOCSY). STOCSY is a valuable statistical tool contributing to the biomarker identification process and was employed for the first time in EVOO analysis. Market samples from three Mediterranean countries of Spain, Italy, and Greece, blended samples from these countries, as well as monocultivar samples from Greece were analyzed. The NMR spectra were collected, with the help of chemometrics acting as “fingerprints” leading to the discovery of certain chemical classes and single biomarkers that were related to the classification of the samples into groups based on their origin.

## 1. Introduction

Extra virgin olive oil (EVOO) is a core element of the Mediterranean diet due to its nutritional, health, and sensory traits [1,2]. According to the European Commission, there are eight different types or categories of olive oil, graded based on their quality parameters related to physicochemical characteristics, such as acidity level, peroxide index, fatty acid content and sterols composition, and organoleptic (sensory) characteristics, such as the fruitiness and the absence of organoleptic defects [3]. The highest-rated, EVOO is often the target of fraudulent practices, such as its adulteration with lower quality olive oils or inexpensive edible vegetable oils and mislabeling by a false declaration of origin [4]. Based on these grounds, guidelines and standards have been issued by the International Olive Council (IOC), Codex Alimentarius and European Commission regarding requirements of commercial categories and marketing standards, as well as providing analytical methods that would ensure quality and authenticity [5,6,7,8,9]. Particularly in the case of the European Union (EU), a quality policy, aiming to protect the names of specific products and promote their unique characteristics linked to their geographical origin, as well as traditional know-how, has been established [10]. Protected Designation of Origin (PDO) and Protected Geographical Indication (PGI) are two labels commonly attached to EVOOs. As of September 2020, almost 140 EVOOs from Spain, Italy, Greece, France, Portugal, Croatia, and Slovenia have been accepted into the PDO and PGI registry [11].

Aiming to affirm and protect the quality and authenticity of EVOOs, conventional analysis has been applied to this day. In particular, free acidity, iodine value, sterols, biophenol content, fatty acid composition, and positional distribution of the glycerol moiety are analyzed. Additionally, guidelines have been issued regarding their sensory analysis [12]. Speaking of authenticity concerning EVOO, it is usually associated with geographical origin, genetic variety, and quality grade [4]. In the case of geographical origin, numerous studies have been conducted on regional [13,14], national [15,16], Mediterranean/international [17,18,19], and PDO/PGI scales [20,21,22] employing NMR spectroscopy and LC- or GC-MS. Specifically, concerning NMR spectroscopy, different nuclei have been recruited, with ^1^H, ^13^C, and ^31^P being the most common ones [16,17,22]. Monocultivar samples have been analyzed with regard to the variety aspect, mainly from Italy and Greece [23,24,25,26].

Nevertheless, investigating the geographical origin of oils from the top three producing countries worldwide has been the subject of very few studies [27]. The majority of the time, metabolite fingerprinting with ^1^H NMR has been utilized for the classification of EVOOs [28], followed by aroma/volatiles profiling with GC-MS [19]. To our knowledge, only Olmo-García et al., have searched for geographical discriminative biomarkers between oils from Spain, Italy, and Greece with PDO and PGI labeling, which was accomplished both with GC-MS and LC-MS [18].

Furthermore, a typical challenge in such studies regardless of the type of sample e.g., EVOO, virgin olive oil (VOO), PDO, and/or the goal, e.g., geographical or botanical origin, is the annotation of any revealed biomarker [29]. Commonly, classification could be achieved to a certain degree; however, the identification of statistically significant features remains highly uncertain [30]. Different tools have been suggested depending on the analytical method using most of the times chemical and/or spectral databases, which are, however, incomplete, specific, or random, especially in food components or bioactives [31]. In NMR spectroscopy in particular, a tool that could aid in the identification procedure is statistical total correlation spectroscopy, or STOCSY, which was first introduced in the study of biological samples. It generates a pseudo-spectrum by correlating peaks with the same fluctuation across NMR spectra of the respective samples by calculating the correlation coefficient between a defined “driver” peak and all other signals in the spectrum [32].

Therefore, the aim of the current study was the development and application of an NMR-based metabolite profiling method that would enable the mapping of commercial EVOOs from Spain, Italy, and Greece. Chemometrics were then employed for the identification of certain marker compounds. STOCSY, a strong statistical tool, was used for the first time in EVOO analysis to aid in the identification process [33,34]. Additionally, two Greek cultivars, Koroneiki and Kolovi, obtained directly from producers, were also examined for the first time with ^1^H NMR metabolite profiling. Finally, dereplication on the ^1^H NMR spectrum of the total extract of olive oil was attempted using STOCSY pseudo-spectra, 2D-recorded spectra of different total extracts, and the available literature.

## 2. Results and Discussion

The quality of EVOO, aside from its age and sensory and nutritional properties, encapsulates the concept of authentication and fraudulence. For better differentiation, Dais and Hatzakis designated three categories, i.e., adulteration with foreign oils and fraud regarding geographical and botanical origin [35]. A plethora of metabolomics approaches, both targeted and non-targeted, have been used to investigate these attributes in EVOO by measuring classes of chemical components such as organic acids, sterols, TAGs, pigments, volatiles, and phenols [24,27,36,37]. The current study focused on the latter two aspects, the topography and cultivar of the tree from which the produced oil originated. Moreover, it concentrated on NMR-based metabolite profiling of two sets of samples, i.e., EU market EVOOs and monocultivars from Greece, reaching over 240 samples.

### 2.1. Sample Selection and Preparation

EVOO samples were carefully selected in a representative way. For the first set comprised market samples from Europe, the top three countries in average production for the period 1993–2014 [38], Spain, Italy, and Greece, were selected. Additionally, several blends from these countries and the extended European region were included. These samples were commercial and, in most cases, no cultivar was known, which could translate to multi-cultivar oils. Therefore, only the geographical origin was examined in this sample set. Details about the origin of these samples can be found in Table S1 and can be summarized as follows: 29 from Spain, 29 from Greece, 19 from Italy, 11 blends from these three countries, and 22 labeled as blends with EU origin.

A different approach was followed for the second set comprised oils from Greece. Monocultivar EVOOs were collected from producers dispersed all over Greece. Koroneiki, being the predominant variety cultivated in Greece [39], was selected for this study, along with Kolovi and other mainly local varieties such as Thiaki, Adramitiani, Manaki, and Megaritiki. As far as the geographical origin is concerned, Crete and Peloponnese, responsible for most of the country’s production, were included together with the island of Lesvos from the North Aegean region. Samples from the Ionian Islands were excluded from further statistical processing due to their inadequate number and high diversity (four samples from three different varieties) that could not produce a reliable outcome. Similarly, the only sample belonging to the Adramitiani variety was from Lesvos, and samples from Argolida and Lakonia were also excluded either due to a lack of complete metadata or a lack of an adequate number of samples. Consequently, a total number of 121 samples from the second set were examined further. Details about the geographical and botanical origin of the samples can be found in Table S2. It is important to note that special attention was given to the collection and the quality of metadata accompanying each sample in order to be qualified for further analysis. The high variability and complexity of such samples could lead to vague and misleading results if data are incomplete and/or not trustful. All samples were centrifuged, stored, preserved, and extracted under the same conditions to minimize any induced variability. Regarding the extraction protocol, the method of the IOC was adapted based on the sensitivity of the analytical platform selected here, NMR spectroscopy [40].

### 2.2. Acquisition, Processing, and Multivariate Analysis

In the course of the last two decades, NMR spectroscopy has earned its place as a high-throughput, precise, and quantitative approach for the assessment of adulteration and discrimination of botanical and geographical origin of foods [41]. To that end, this platform opted to analyze a total of 241 EVOO samples from Europe and Greece. One dimensional spectra of all samples were acquired at 600 MHz in chloroform-*d*, preprocessed within the Topspin environment (phase/baseline correction, referencing to internal standard), and processed with the MATLAB suite (alignment, binning, normalization) prior to being subjected to multivariate analysis (MVA).

#### 2.2.1. European Samples

**Figure 1:**
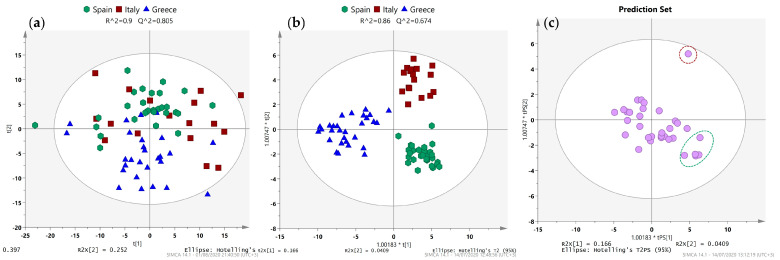
(**a**) PCA scores scatter plot of PC1 and PC2 with the European samples examining geographical origin; (**b**) OPLS-DA scores scatter plot depicting the separation between Spain, Italy, and Greece; (**c**) scores scatter plot of all blended samples as the prediction set. One sample appears to contain mainly Italian EVOOs, five mainly Spanish, and the rest lie among the three countries.

Samples of this group were mainly multicultivar and produced in Spain, Italy, Greece, and the extended European region. Quantitative variations can be observed in the spectra of the three countries, but qualitative differences are not that apparent (Figure S1). To initially detect any potential trends within the data, principal component analysis (PCA), an unsupervised approach, was selected. The resulting model comprised 10 principal components accounting for 90.0% of the total variance in the dataset. Looking at the scores scatter plot of the first two components (Figure 1a), PC1 and PC2, a reasonable separation of Greek samples is observed, while samples from Italy and Spain exhibit significant overlap using this unsupervised approach.

Partial least squares discriminant analysis (PLS-DA) and orthogonal projections to latent structures discriminant analysis (OPLS-DA) models were then constructed in a supervised manner aiming to find potential markers that could differentiate samples from these countries. The scores scatter plot of the PLS-DA model already presents a clearer separation, with samples from Italy and Spain still being intertwined (Figure S2a). Permutation tests verified the validity of the model aside from the R2Y and Q2 values (Figure S2b–d) to be of good predictive quality. However, the OPLS-DA model constructed with samples from all three countries allows excellent discrimination of the countries of origin and was used to assess the composition of the blended European samples. In detail, clear grouping was observed, with Greek samples being separated from the rest by the first predictive X-Y component and Italian with Spanish by the second (Figure 1b). The scores scatter plot of blended samples placed as a prediction set in the aforementioned model recognized one sample that supposedly contained mainly EVOOs of Italian origin and five of Spanish (Figure 1c). The remaining samples lay in between and thus could indeed be confirmed as blends.

Moreover, three OPLS-DA models were constructed with countries taken in pairs as part of the workflow to detect statistically significant compounds from the different regions (Figure S3) by examining the variable importance for the projection (VIP) values and correlation coefficients [p(corr)] of the variables.

#### 2.2.2. Greek Samples

**Figure 2:**
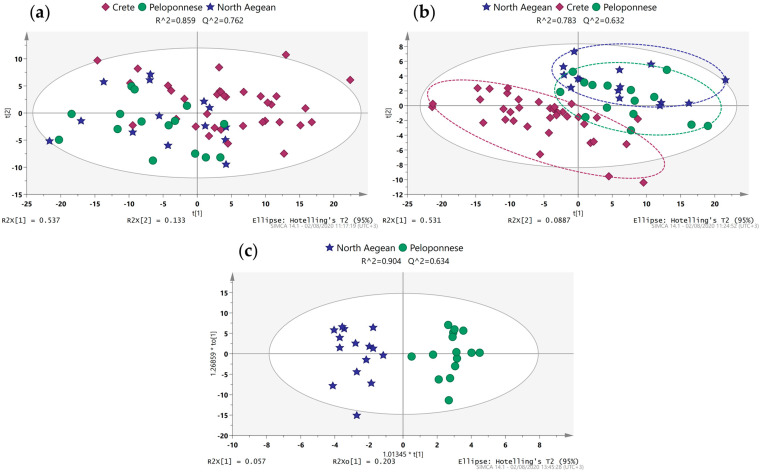
(**a**) Unsupervised PCA scores scatter plot of PC1 and PC2 with the Greek samples examining geographical origin; (**b**) respective supervised PLS-DA scores scatter plot; (**c**) OPLS-DA scores scatter plot with samples from the North Aegean vs. Peloponnese.

Samples included in this section are dispersed over three regions of Greece, including Crete, Peloponnese, and Lesvos, with the majority belonging to the Koroneiki variety and fewer to Kolovi. Initially, the parameter of geographical origin was explored using only monocultivar samples. Under no supervision, slight trends were detected in the PCA scores scatter plot defined by the first two components (Figure 2a). Samples from Crete seemed to differ to a larger extent from the other two classes, which were greatly entangled. The PLS-DA scores scatter plot of the primary two components presented a tighter grouping of the classes, with samples from the North Aegean and Peloponnese still being entangled to a great extent (Figure 2b). Finally, OPLS-DA models offered the possibility of a one-to-one comparison among regions. To that end, three scores scatter plots were created in order to provide visualization of their respective models (Figure 2c and Figure S5). Tight clustering along with good fitting and predictability values (R2Y and Q2) was observed in all three models, as also verified by their respective CV scores scatter plots (Figure S5).

**Figure 3:**
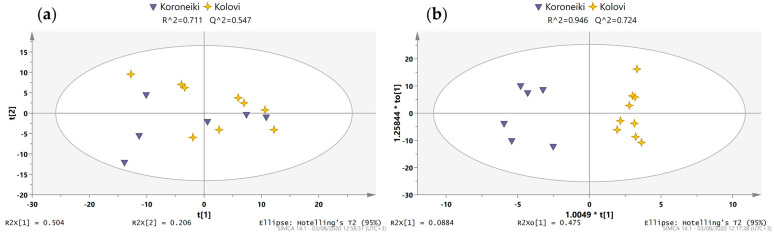
(**a**) PCA scores scatter plot of PC1 and PC2 with the Greek samples examining botanical origin; (**b**) respective OPLS-DA scores scatter plot.

In the study of the variety parameter, only Koroneiki and Kolovi could be examined, as they were the only ones from which enough samples were collected to provide a statistically meaningful outcome. Furthermore, samples only from the North Aegean were examined to ensure that any variation in the samples was due to the different variety and not the region. These varieties were examined for the first time with ^1^H NMR metabolite profiling, as only two previous studies have implemented ^31^P NMR and LC-MS profiling [25,26]. The built PCA model formed by two principal components with R2X and Q2 at 0.711 and 0.547 respectively, revealed a reasonable trend among the two varieties (Figure 3a). In the OPLS-DA model, the two classes were separated clearly in the predictive component (*x*-axis) (Figure 3b), resulting in a model with great goodness of fit (0.946) and predictability (0.724). A two-way filtering process, as with samples from Europe, was applied for the extraction of a tentative biomarkers list. The final list contained features filtered based on their VIP and p(corr) values (Table S4).

### 2.3. Statistical Total Correlation Spectroscopy (STOCSY) and Biomarker Identification

Untargeted metabolomics can aid in the mining of spectral features with statistical significance. The difficulty, however, lies in their correlation with distinct compounds that have a possible link to certain quality attributes. In NMR spectroscopy, this is often additionally complicated due to the lack of standardized high-quality databases and the extended peak overlap. In this study, a three-part approach was invoked in an attempt to assign as many peaks as possible in the 1D spectrum of an EVOO total extract. STOCSY, a very strong statistical tool applied for the first time in olive oil analysis, was combined with 2D spectra (JRES, COSY, HSQC-DEPT, HMBC) of selected extracts and the combination of an in-house spectral database and the literature.

**Figure 4:**
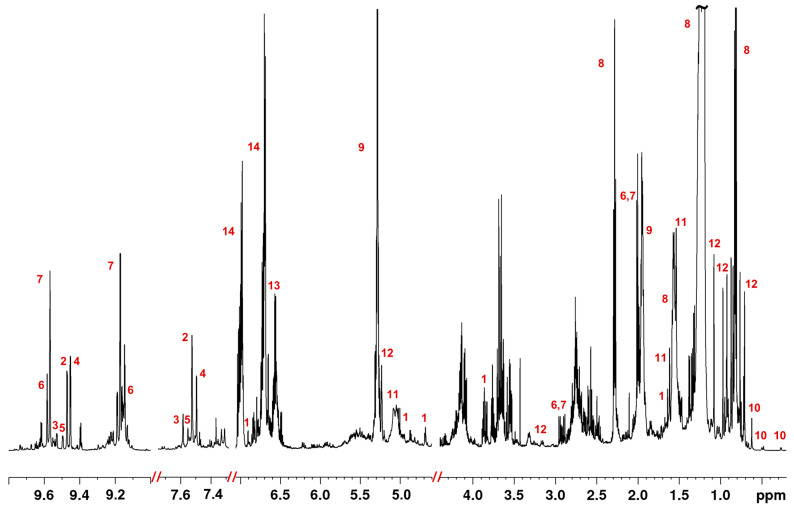
Annotated representative 1D 1H NMR spectrum of a Greek EVOO (600 MHz, chloroform-*d*, reference with IS at 0 ppm). 1: Acetoxypinoresinol; 2: monoaldehydic form of oleuropein aglycon (MFOA)-5S,8R,9S isomer; 3: MFOA-5S,8S,9S isomer; 4: monoaldehydic form of ligstroside aglycon (MFLA)-5S,8R,9S isomer; 5: MFLA-5S,8S,9S isomer; 6: oleacein; 7: oleocanthal; 8: all fatty acids; 9: unsaturated fatty acids; 10: sterols; 11: squalene; 12: triterpenes; 13: hydroxytyrosol and derivatives; 14: tyrosol and derivatives.

As could be easily observed in Figure 4, the protons of olive oil’s polar constituents (total phenolic fraction or TPF, see Section 3.2) resonate in certain characteristic spectral regions, indicative of different spin systems. Probably, the most characteristic region is at the low field, where the peaks of aldehydes groups of mainly secoiridoids, such as monoaldehydics, form oleuropein aglycon (MFOA), and monoaldehydic forms of ligstroside aglycon (MFLA), oleacein, and oleocanthal appear. Additionally, typical is the aromatic region where the protons of tyrosol and hydroxytyrosol, as well as their derivatives, resonate. It is worth noting that these secoiridoids commonly comprise the major biophenols of olive oil and have been suggested as authentic and generally quality markers of olive oil [42,43]. The annotation of the compounds was based on the literature, but also the use of reference standards.

Along these lines, toward revealing biomarkers and at the same time increasing the identification confidence, STOCSY was employed. STOCSY takes advantage of collinear gradients of peaks to generate pseudo-spectra reflecting the actual ^1^H NMR spectrum of the targeted compound. Even if it is used in human metabolomics [32], its employment in food analysis and generally in natural products is limited. To our knowledge, it has been used in the study of medicinal plants [44,45,46,47], whisky [48], and table olives and honey samples from our group [33,34].

**Figure 5:**
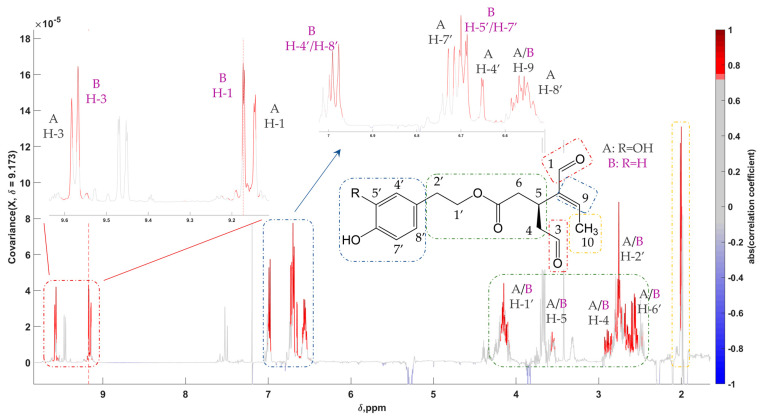
Statistical total correlation spectroscopy (STOCSY) 1D pseudo-NMR spectrum of oleacein (**A**) and oleocanthal (**B**). Correlation coefficients to the other signals in the median EVOO NMR spectrum are color-encoded: the “driver peak” was at 9.173 ppm. Zoom-in of the aldehydic and aromatic regions is also presented.

Initially, the focus turned to two doublet peaks at around 2.0 ppm with a high VIP value of approximately 1.5 in favor of Cretan oils in the Greek sample set. Employing STOCSY, a series of other peaks were revealed, leading to the final use of the resonance at 9.173 to obtain a clearer pseudo-spectrum (Figure 5). In that way, two compounds were discovered, oleacein and oleocanthal, two secoiridoids generally present in high concentrations in EVOOs [49]. Due to their proportional fluctuation in their concentration levels among samples, both compounds appear in one pseudo-spectrum with the same driver peak. Complete assignment of the peaks in the total extract was accomplished with the aid of 2D spectra (Figures S6 and S7), reference standards, and the literature, i.e., the recent study by Sarikaki et al. involving the aforementioned compounds [50].

**Figure 6:**
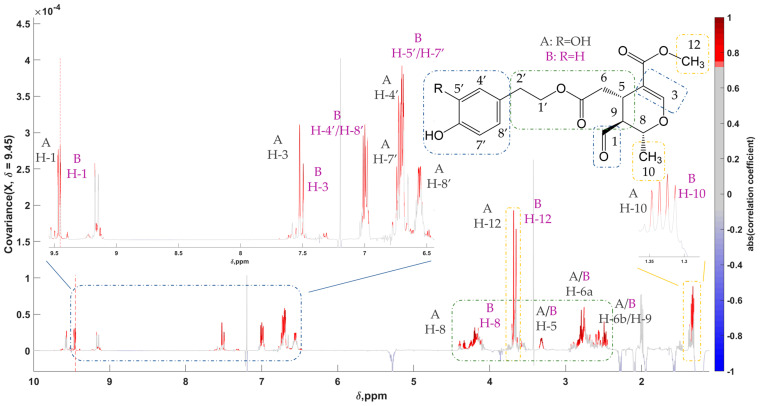
STOCSY 1D pseudo-NMR spectrum of MFOA-5S,8R,9S isomer (**A**) and MFLA-5S,8R,9S isomer (**B**). Correlation coefficients to the other signals in the median EVOO NMR spectrum are color-encoded: the “driver peak” was at 9.450 ppm. Zoom-in of the aldehydic and aromatic regions is also presented.

Furthermore, an analogous behavior was observed in the case of the monoaldehydic form of oleuropein and ligstroside aglycons, also known as MFOA and MFLA, respectively. In EVOO, a mixture of two diastereoisomers can be detected, 5S,8R,9S, and 5S,8S,9S, with the former found usually in higher concentrations [49,51]. A single 1D STOCSY was generated with the most abundant isomers of the two with the driver peak set at 9.450 ppm (Figure 6). The research of both isomers of MFOA and MFLA that were successfully isolated by Angelis et al., in addition to acquired 2D spectra, proved to be a valuable aid in assigning many of their peaks across the total extract spectrum (Table S5) [49]. Moreover, in the aromatic regions, the plethora of overlapping peaks, among which the ones of oleacein, oleocanthal, MFOA, and MFLA, were deciphered by an earlier study [52]. The multi-suppression pulse sequence used over the lipids allowed the direct measurement of the phenols contained in olive oil without prior treatment. Peaks resonating in the region between 6.7 and 7.1 ppm were attributed to tyrosol, hydroxytyrosol, and their derivatives containing one of the two moieties, respectively. Discrimination between the two groups of compounds was accomplished through the variability in coupling constants. As for the group with the tyrosol moiety, it was found at 8.5 Hz for aromatic protons with just an ortho coupling, whereas the same coupling was found to be 8.0 Hz for the hydroxytyrosol moiety.

**Figure 7:**
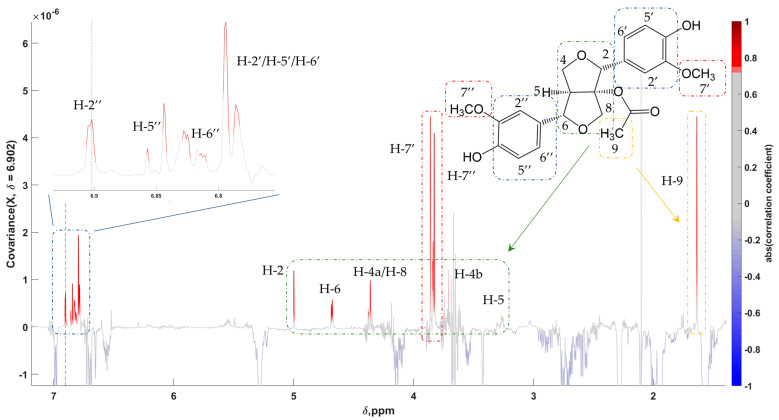
STOCSY 1D pseudo-NMR spectrum of acetoxypinoresinol. Correlation coefficients to the other signals in the median EVOO NMR spectrum are color-encoded: the “driver peak” was at 6.902 ppm. Zoom-in of the aromatic regions is also presented.

The lignan acetoxypinoresinol was primarily spotted by feature 44 from the OPLS-DA model for differentiating samples from Spain vs. Greece. The singlet resonating at 3.86 ppm could be assigned to the protons of one of the two methoxy groups of the molecule. The pseudo-spectrum created with 6.902 ppm as the driver peak, aside from revealing a clear representation of acetoxypinoresinol with almost all peaks shown (Figure 7), discovered several anticorrelations, such as SFAs and unsaturated fatty acids (UFAs) resonating at around 1.20, 1.95, and 5.30 ppm, as well as phenolic compounds with the tyrosol and hydroxytyrosol moiety (6.7–7.1 ppm). This observation could also be useful as it indicates the opposite fluctuation of the levels of the anti-correlated compounds between the examined groups.

Olive oil is also an important source of another significant class of compounds, triterpenes. Oleanolic, maslinic, and ursolic acids, along with the alcohols uvaol and erythrodiol, are among the most common in this chemical category [52,53]. In this case, the resonance at 0.706 ppm, a tentative biomarker in two out of three OPLS-DA plots of the European sample set in favor of samples from Greece (Table S3), was used as a driver peak. The constructed STOCSY (Figure S8) turned out to reveal several methyl protons between 0.7 and 1.2 ppm, all belonging to the above-mentioned compounds. Due to their structural resemblance, discrimination between them was not possible. Squalene, a non-pentacyclic terpenoid, was identified with the aid of STOCSY and the literature [54]. A singlet peak at 1.61 ppm produced a pseudo-spectrum with all signals of the compound being present (Figure S9). Unfortunately, another compound presented a similar fluctuation in its concentration, hence appearing in the same spectrum.

Signals of sterols were detected in a region that is free of other resonances, between 0.2 and 0.7 ppm. Specifically, cycloartenol, cyclobranol, and 24-methylenecycloartanol were responsible for two doublet peaks at 0.26 and 0.49 ppm, while gramisterol, citrostadienol, Δ7-avenasterol, and Δ7-campesterol were detected by a singlet at 0.48 ppm. Resonances from the former sterols were determined as statistically significant based on their VIP and p(corr) values in OPLS-DA models for Italian samples, while the latter were in favor of samples from Greece. After a literature search, an assignment in this chemical category was completed for β-sitosterol, Δ5-avenasterol, and Δ5-campesterol, as their methyl protons at C-18 resonated at 0.61 ppm as a singlet [52,55].

Finally, FAs, the main chemical group in EVOOs, were investigated. Despite the extraction, a complete removal is impossible with the IOC-suggested method. An indicative 1D pseudo-spectrum was generated with a driver peak at 1.19 ppm. Acyl groups of both SFA and UFA chains could easily be detected [52,56] (Figure S8). A review by Alexandri et al. proved critical in identifying peaks that belonged to differently substituted forms of glycerol, MAG, 1,2 and 1,3-DAG, and TAG [57].

Still, several statistically significant features remain unidentified in particular in the Greek sample set. To be more precise, using spectral feature 2 listed in Table S4 from the OPLS-DA model for discriminating the North Aegean and Peloponnese regions of Greece, a clear pseudo-spectrum was generated (Figure S10). Interestingly enough, the resonances of all peaks, belonging to most probably two compounds, can be found in the aforementioned table of tentative biomarkers. Some of them were also extracted from the OPLS-DA plot between Koroneiki vs. Kolovi varieties from the North Aegean region. Therefore, while geographical region influences the presence and concentration of said biomarkers, it seems that they are also affected to some extent by the variety parameter. Hence, it must be concluded that the classification of geographic origins in part is influenced by the variety, which may be considered a confounding effect.

### 2.4. Quality and Authentication Assessment

Olive oil has been extensively investigated over the years regarding its quality attributes. Aside from the established organoleptic assessment through sensory analysis by a testing panel [58], authenticity falls within the margins of quality. Generally, it envelopes genetic variety, geographical origin, and quality grade, including adulteration with lower-quality olive oils or vegetable oils. In the current study, attention was given to the identification of specific markers or patterns indicative of different geographical origins and cultivars. Specifically, through metabolite profiling, traceability from top-producing countries of Europe, and different regions from Greece, two different cultivars were examined.

**Figure 8:**
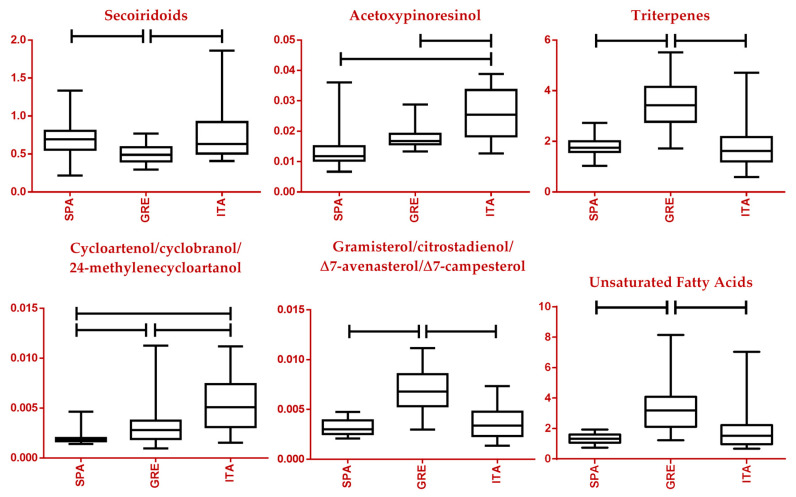
Box plots of a selection of statistically significant markers in the European sample set. Specifically, Secoiridoids, Acetoxypinoresinol, Terpenoids, Cycloartenol/Cyclobranol/24-methylenecycloartanol, Gramisterol/Citrostadienol/Δ7-Avenasterol/Δ7-Campesterol, and UFAs are depicted (vertical axis expressed in normalized intensity). Horizontal lines between classes indicate a *p*-value lower than 0.05. SPA: Spain, GRE: Greece, ITA: Italy.

**Figure 9:**
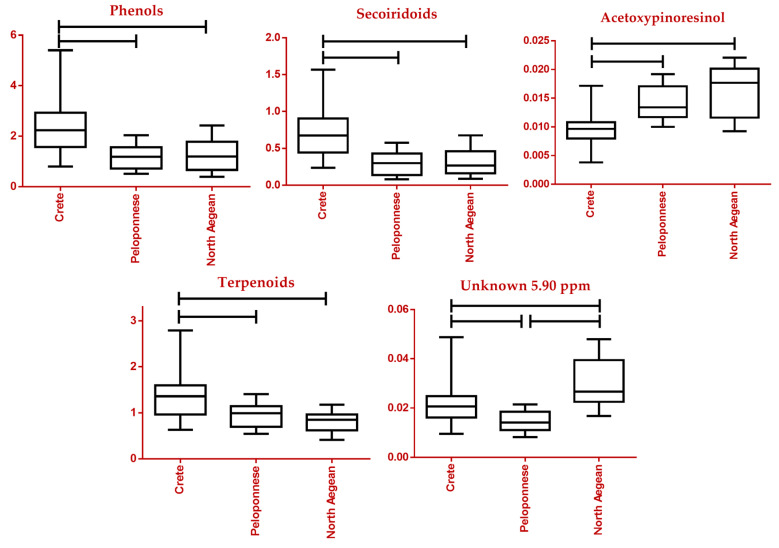
Box plots of a selection of statistically significant markers in the Greek sample set. Specifically, Phenols, Secoiridoids, Acetoxypinoresinol, Terpenoids, and an unknown tentative marker are depicted (vertical axis expressed in normalized intensity). Horizontal lines between classes indicate a *p*-value lower than 0.05.

Adhering to the designed workflow, identified and statistically significant biomarkers drew attention, as their relative concentration in the different classes was monitored. Primarily, two major classes of compounds were investigated, secoiridoids and phenols. As can be seen in the respective box plots, it seems that Greek EVOOs exhibited lower concentrations in secoiridoids compared to samples from Italy and Spain, while no statistically significant difference was observed when it came to phenols (Figure 8 and Figure S11). These compounds have not been examined in terms of chemical categories as a whole and, therefore, no comparisons to the literature are feasible. As far as the Greek sample set is concerned, samples from the island of Crete outmatch both other regions, the Peloponnese and North Aegean, in total phenols and secoiridoids, as well as in oleocanthal content, a secoiridoid indicated as a tentative biomarker revealed by the MVA performed (Figure 9 and Figure S12). This observation was verified by previously conducted studies [16].

The lignans of EVOO have been reported to possess anti-inflammatory, antitumor, antioxidant, and neuroprotective activities [59]. Among the two major ones is acetoxypinoresinol. Italian samples appeared to be significantly richer in this compound, with Spanish and Greek samples displaying approximately equal levels. Again, no studies involving this compound and these countries have been previously conducted to our knowledge. In the case of the Greek sample set, samples from both the North Aegean and Peloponnese outweighed the Cretan ones (Figure 9), contrary to previous research where Crete surpassed Peloponnese [16].

A different pattern is observed in the case of triterpenes, which are found in various organs and products of the olive tree [60]. Triterpenic acids and especially maslinic and oleanolic acids have important activity profiles such as antitumor, antidiabetic, antioxidant, cardioprotective, antiparasitic, and neuroprotective [61,62]. The scales were in favor of Greek EVOOs with an almost double relative concentration when compared to Spain and Italy (Figure 8). Previous studies, however, cannot seem to come to a firm conclusion. On one hand, Mannina et al. described the edge of olive oils from the Liguria province of Italy over oils from other European countries, among them Spain and Greece [63]. Another study conducted in 2019, exploring the content of commercial PDO and PGI EVOOs, finds higher contents in Greek samples as compared to Spanish and Italian ones, corroborating our results [18]. Regarding the Greek sample set, triterpene content for samples from Peloponnese and North Aegean is underwhelming as opposed to Crete (Figure 9).

An interesting remark can be made when these results are compared with a recent study of our group concerning metabolite profiling of EVOO using an FIA-MRMS platform [42]. It seems that both studies agreed with regard to the concentration of several compounds or classes. Specifically, samples from Crete were found to be superior to the ones from the Peloponnese in secoiridoids, oleocanthal especially, and also triterpenes. On the other hand, acetoxypinoresinol presented the opposite pattern, corroborating our findings.

While sterols resemble minor compounds in olive oil, they seem to be greatly affected by the geographical coordinates of the cultivation site [64]. This was also observed in the current study. Samples from the Italian peninsula appeared to be rich in cycloartenol/cyclobranol/24-methylenecycloartanol, followed in order by Greek and Spanish ones. Nevertheless, a subversive image is discerned with gramisterol/citrostadienol/Δ7-avenasterol/Δ7-campesterol, where Greece outweighed by a great margin the remaining two countries (Figure 8). These observations are in accordance with past studies [18,55]. No statistical significance was attributed to sterols for any of the classifications in the Greek set of samples.

A very important chapter in olive oil analysis is FAs, both saturated and unsaturated ones. Among the three European countries investigated in this study, Greece led Spain and Italy in both categories, as can be seen in the respective box plots (Figure 8 and Figure S11). Despite the importance of this class of compounds, past research has focused on the study of geographical origin on a regional scale with no studies conducted to our knowledge between the three top-producing countries [13,22,24,65]. Moving on to monocultivar samples from Greek producers, it seems the different regions present a similar profile in terms of SFA and UFA content, with no statistical significance in any observed variations (Figure S12). A similar observation has been made for samples from the Peloponnese and Crete [16].

The tentative biomarker, revealed initially from the statistically significant features from the OPLS-DA models of the Greek sample set with a peak resonating at 5.90 ppm (Figure S10), is of great interest. High fluctuation in its concentration was observed depending on the geographical origin of the samples. Linoleic acid, a tentative biomarker of the Kolovi variety, was examined and validated for its statistical importance (*p*-value < 0.05). One previous study sought markers for the discrimination of EVOOs from Greek cultivars, among them Kolovi and Koroneiki, but linoleic acid was not among the four that were detected [26]. Further studies are needed to identify unknown compounds and further explore the effect of geographical origin and cultivar in the composition of EVOOs.

## 3. Materials and Methods

### 3.1. Chemical and Materials

#### 3.1.1. Solvents and Reference Compounds

Solvents used for extraction of the oils, MeOH and H_2_O, were of HPLC grade (Fisher Scientific, Loughborough, UK). The deuterated NMR solvent used, chloroform-*d* (purity 99.8% D), was acquired from Eurisotop GmbH (Saarbrücken, Germany). Hexamethyldisiloxane (HMDSO; NMR grade, ≥99.5%, Sigma-Aldrich Corporation, St. Louis, MO, USA) was used as a line-shape indicator. NMR tubes (D600-5-7, 5 mm diameter and 7 inches long) with PTFE caps were obtained by Deutero GmbH (Kastellaun, Germany).

#### 3.1.2. Sample Selection

Two sets of EVOO samples were selected for this study. The first, comprising 110 samples, was commercially available (processed and bottled) samples obtained from European cooperatives during the harvesting period 2017–2018. They originated from countries in Europe. For the second set, obtained directly from producers during the harvesting period 2018–2019, a total of 131 EVOOs were analyzed originating from different areas of Greece and different cultivars. Upon receipt, samples were stored under a nitrogen atmosphere in dark glass vials at 20 °C to maintain their chemical stability. Details about the samples are listed in Tables S1 and S2.

#### 3.1.3. Instrumentation

A Bruker AVANCE III 600 NMR spectrometer (Bruker BioSpin GmbH, Rheinstetten, Germany), operating at the proton frequency of 600.13 MHz (B0 = 14.1 T) and carbon frequency of 150.91 MHz and equipped with a 5 mm broadband probe with inverse detection (BBI), and *z*-axis gradients was used for the recording of the acquisition of the spectra. A Bruker Cooling Unit (BCU) was used to stabilize the temperature. Spectra were recorded with the help of a 60-place sample changer (B-ACS-60) using the IconNMR automation software (version 5.0) by Bruker. Topspin software (version 3.5.7 and 4.0.6) by Bruker was used for the acquisition and processing of the spectra.

### 3.2. Sample Extraction

Extraction was carried out by employing a modified version of the IOC protocol [40]. A total of 7.0 g of EVOO were weighed in a 50 mL screw-cap falcon and 21 mL of extractant solution was added (MeOH/H_2_O: 80/20 *v*/*v*). The mixture was vortexed for 1.5 min and placed for 15 min in an ultrasonic bath at room temperature. Then, it was centrifuged at 4000× *g* rpm for 25 min and the supernatant phase was passed through a 0.45 µm PVDF filter. A total of 15 mL of the recovered phase was transferred in a spherical flask and evaporated to dryness with a RotaVapor at 40 °C under a vacuum (Büchi AG, Flawil, Switzerland). Reconstitution was accomplished with MeOH in order to transfer the extract to an Eppendorf tube. Finally, it was evaporated to dryness using a centrifugal evaporator with a vacuum (Concentrator Plus, Eppendorf AG, Hamburg, Germany) and stored at −20 °C, pending analysis.

### 3.3. NMR Analysis

#### 3.3.1. Sample Preparation

A stock solution of chloroform-*d* with 0.02% *v*/*v* HMDSO was prepared. Each sample was dissolved in 650 µL of the stock solution and 600 µL was transferred into an NMR tube.

#### 3.3.2. NMR Experimental Parameters

Spectra were recorded at 305 K with a simple “zg” pulse sequence with the following conditions: ns, 64; π/2 pulse, ~8 μs; TD, 64 k data points; acquisition time, 3.89 s; relaxation delay, 2.00 s; and spectral width, 8417.5 Hz. The spectra were obtained by the Fourier transformation (FT) of the free induction decay (FID) with the application of an exponential function using a line-broadening factor of 0.3 Hz and zero-filling (size = 128 k). The resulting spectra were manually phased and baseline-corrected using a polynomial function in the TopSpin software (version 4.0.6). Chemical shifts were reported based on the IS’s signal, which was set at 0.0 ppm. Two dimensional spectra (COSY, HSQC-DEPT, HMBC, and JRES) were acquired based on common pulse sequences containing gradients from the Bruker library (cosygpqf, hsqcedetgpsisp2.3, hmbcetgpl2nd, and jresgpqf, respectively).

### 3.4. Computational Processing and Multivariate Analysis

The MATLAB suite (version R2018b) was used for the alignment of the spectra with the icoshift tool, and a targeted selection of intervals optimized for these samples (Version 3.0) [66]. Then, NMR spectra were binned between −0.5 and 12 ppm, with a bin width of 0.01 ppm, resulting in a total of 1211 bins for MVA or a bin width of 0.001 ppm giving 12,101 bins for STOCSY analysis, respectively. Data were normalized using an in-house routine, with the area of the IS’s peak as a reference value.

For MVA, data were imported into SIMCA v. 14.1 (Umetrics, Umea, Sweden), where they were specifically subjected to PCA, PLS-DA, and OPLS-DA analysis, respectively. Regions with solvent signals (chloroform, methanol), baseline noise, and the IS signal were removed, resulting in 798 features. Prior to PCA, data were scaled using Pareto scaling and subsequently log-transformed.

Sample discrimination was mainly accomplished using the OPLS-DA algorithm and a binary one-vs.-one testing strategy in order to find statistically important distinguishing metabolites for the classes investigated. Variable importance for the projection (VIP) lists generated for each model ranked variables based on their contribution to the model. Variables with a score greater than 1 were considered statistically significant. Generated models were evaluated for the goodness of fit and predictability, described by their R2X/R2Y and Q2 values, respectively. Only models with R^2^ and Q^2^ values above 0.5 and close to each other were accepted. Permutation tests (500 permutations) and CV scores scatter plots (7 rounds performed) were also employed to further validate the models. Outliers were excluded using Hotelling’s T2, DModX, and DModY plots.

One dimensional STOCSY analysis by exploiting the data with the 0.001 ppm bin size was carried out with an in-house routine in the MATLAB suite [33,34]. A threshold of 0.75 for the correlation coefficient was set on the whole dataset and it was kept at the same level for all generated pseudo-spectra.

## 4. Conclusions

In the present study, an NMR-based workflow is proposed for the authentication and quality determination of EVOO. Metabolite profiling and chemometrics together with STOCSY were applied in two different sets of EVOO samples, one mixed European and one Greek. This was the first application of this method on commercial samples from the three highest-ranking countries in olive oil production worldwide focusing on a broader range of putative marker compounds, including in particular biophenols. In fact, several biomarker compounds were finally identified for discrimination regarding geographical origin. Moreover, both geographical origin and cultivar parameters were investigated in Greek EVOOs from three regions and two varieties. STOCSY, a statistical tool with only a handful of applications in natural products, was successfully incorporated for the first time in olive oil analysis as a dereplication and annotation tool, and also in the attempt to seek statistically significant compounds. The revealing of key peak correlations along with acquired 2D spectra and a widespread literature search led to the identification of numerous compounds in the ^1^H NMR spectrum of EVOO. Finally, certain compounds were suggested as quality markers for commercial EU countries, as well as for different geographical areas of Greece.

## Data Availability

Not applicable.

## Supplementary Materials

The following supporting information can be downloaded at https://www.mdpi.com/article/10.3390/molecules28041738/s1, Figure S1: Multiple displays of spectra from Spain, Italy, and Greece. Quantitative variations are mainly observed across the field; Figure S2: (a) PLS-DA scores scatter plot of the European sample set with a clear distinction of samples from Greece; (b) permutation test with 500 permutations performed at the presented PLS-DA model with samples from Spain; (c) respective permutation test with samples from Italy; (d) respective permutation test with samples from Greece; Figure S3: (a) OPLS-DA scores scatter plot with samples from Spain vs. Italy; (b) indicative CV scores scatter plot from the OPLS-DA model of Spain vs. Italy; (c) indicative S-plot from the OPLS-DA model of Spain vs. Italy; (d) indicative coefficient plot from OPLS-DA model of Spain vs. Italy; (e) OPLS-DA scores scatter plot with samples from Spain vs. Greece; (f) OPLS-DA scores scatter plot with samples from Italy vs. Greece; Figure S4: (a) permutation test with 500 permutations performed for PLS-DA model in Figure 2 with samples from Crete; (b) respective permutation test with samples from the North Aegean; (c) respective permutation test with samples from the Peloponnese; Figure S5: (a) OPLS-DA scores scatter plot with samples from Crete vs. North Aegean; (b) CV scores scatter plot from the OPLS-DA model of Crete vs. North Aegean; (c) OPLS-DA scores scatter plot with samples from Crete vs. Peloponnese; (d) CV scores scatter plot from the OPLS-DA model of Crete vs. Peloponnese; (e) CV scores scatter plot from the OPLS-DA model of North Aegean vs. Peloponnese; Figure S6: Representative JRES spectrum of a Greek EVOO; Figure S7: Representative 2D spectra of a Greek EVOO. (a) COSY; (b) HSQC-DEPT; (c) HMBC; Figure S8: STOCSY 1D pseudo-NMR spectra. Correlation coefficients to the other signals in the median olive oil NMR spectrum are color-encoded. (a) Triterpenes: the “driver peak” was at 0.706 ppm; (b) fatty acids: the “driver peak” was at 1.190 ppm; Figure S9: STOCSY 1D pseudo-NMR spectrum of squalene. Correlation coefficients to the other signals in the median EVOO NMR spectrum are color-encoded: the “driver peak” was at 1.615 ppm; Figure S10: STOCSY 1D pseudo-NMR spectrum of an unknown biomarker. Correlation coefficients to the other signals in the median olive oil NMR spectrum are color-encoded: the “driver peak” was at 5.907 ppm; Figure S11: Box plots of a selection of statistically significant markers in the European sample set. Specifically, saturated fatty acids (SFAs), squalene, and total phenols are depicted (vertical axis expressed in normalized intensity). SPA: Spain, GRE: Greece, ITA: Italy; Figure S12: Box plots of a selection of statistically significant markers in the Greek sample set. Specifically, oleocanthal, SFAs, and unsaturated fatty acids (UFAs) are depicted (vertical axis expressed in normalized intensity); Table S1: Geographical origin of European samples; Table S2: Geographical and botanical origin of Greek samples; Table S3: Statistically significant markers extracted from the respective OPLS-DA models of European oils. Variable ID (ppm), multiplicity/functional group, VIP, and p(corr) values along with the respective class are presented; Table S4: Statistically significant markers extracted from the respective OPLS-DA models of Greek oils. Variable ID (ppm), multiplicity/functional group, VIP, and p(corr) values along with the respective class are presented; Table S5: 1H NMR chemical shifts and assignments for EVOOs’ metabolites identified. Multiplicity (J in Hz) and functional group are also presented.

## Sample Availability

Samples of the compounds are available from the authors.
