# Supplementary material for: NMR-Based Metabolite Profiling and the Application of STOCSY toward the Quality and Authentication Assessment of European EVOOs
Source: Molecules. 2023 Feb 11;28(4):1738. doi: 10.3390/molecules28041738 (PMC9966212; doi:10.3390/molecules28041738)
Supplement: Supplementary file 1 [file molecules-28-01738-s001.zip › molecules-2211867-supplementary.pdf]

Supplementary Materials

Quality and Authentication Assessment of European EVOOs using NMR-based metabolite profiling and STOCSY

Stavros Beteinakis [1], Anastasia Papachristodoulou [1], Peter Kolb [2], Paul Roesch [2], Stephan Schwarzinger [3], Emmanuel Mikros [4], and Maria Halabalaki [1,*]

[1] Division of Pharmacognosy and Natural Products Chemistry, Department of Pharmacy, National and Kapodistrian University of Athens, Panepistimiopolis, Zografou, 15771, Athens, Greece
[2] NBNC—North Bavarian NMR Centre, University of Bayreuth, Universitätsstraße 30, 95447, and ALNuMed GmbH, Gottfried-Keim-Strasse 60, 95448, Bayreuth, Germany
[3] NBNC—North Bavarian NMR Centre, and ForN – Research Unit for Food Quality, and FLMR – Research Unit for German and European Food Law, University of Bayreuth, Universitätsstraße 30, 95447 Bayreuth, Germany
[4] Division of Pharmaceutical Chemistry, Department of Pharmacy, National and Kapodistrian University of Athens, Panepistimiopolis, Zografou, 15771, Athens, Greece
* Correspondence: mariahal@pharm.uoa.gr

Content

Table S1. Geographical origin of European samples.

Table S2. Geographical and botanical origin of Greek samples.

Figure S1. Multiple display of spectra from Spain, Italy and Greece. Quantitative variations are mainly observed across the field.

Figure S2. (**a**) PLS-DA scores scatter plot of the European sample set with a clear distinction of samples from Greece; (**b**) Permutation test with 500 permutations performed at the presented PLS-DA model with samples from Spain; (**c**) Respective permutation test with samples from Italy; (**d**) Respective permutation test with samples from Greece.

Figure S3. (**a**) OPLS-DA scores scatter plot with samples from Spain vs Italy; (**b**) Indicative CV scores scatter plot from the OPLS-DA model of Spain vs Italy; (**c**) Indicative S-plot from the OPLS-DA model of Spain vs Italy; (**d**) Indicative Coefficients plot from OPLS-DA model of Spain vs Italy; (**e**) OPLS-DA scores scatter plot with samples from Spain vs Greece; (**f**) OPLS-DA scores scatter plot with samples from Italy vs Greece.

Table S3. Statistically significant markers extracted from the respective OPLS-DA models of European oils. Variable ID (ppm), multiplicity/functional group, VIP and p(corr) values along with the respective class are presented.

Figure S4. (**a**) Permutation test with 500 permutations performed for PLS-DA model in Figure 2 with samples from Crete; (**b**) Respective permutation test with samples from North Aegean; (**c**) Respective permutation test with samples from Peloponnese.

Figure S5. (**a**) OPLS-DA scores scatter plot with samples from Crete vs North Aegean; (**b**) CV scores scatter plot from the OPLS-DA model of Crete vs North Aegean; (**c**) OPLS-DA scores scatter plot with samples from Crete vs Peloponnese; (**d**) CV scores scatter plot from the OPLS-DA model of Crete vs Peloponnese; (**e**) CV scores scatter plot from the OPLS-DA model of North Aegean vs Peloponnese.

Table S4. Statistically significant markers extracted from the respective OPLS-DA models of Greek oils. Variable ID (ppm), multiplicity/functional group, VIP and p(corr) values along with the respective class are presented.

Figure S6. Representative JRES spectrum of a Greek EVOO.

Figure S7. Representative 2D spectra of a Greek EVOO. (**a**) COSY; (**b**) HSQC-DEPT; (**c**) HMBC.

Table S5. ^{1}H NMR chemical shifts and assignments for EVOOs' metabolites identified. Multiplicity (J in Hz) and functional group are also presented.

Figure S8. STOCSY 1D pseudo-NMR spectra. Correlation coefficients to the other signals in the median olive oil NMR spectrum are color-encoded. (**a**) Triterpenes: "driver peak" was at 0.706 ppm; (**b**) Fatty acids: "driver peak" was at 1.190 ppm.

Figure S9. STOCSY 1D pseudo-NMR spectrum of squalene. Correlation coefficients to the other signals in the median EVOO NMR spectrum are color-encoded: “driver peak” was at 1.615 ppm.

Figure S10. STOCSY 1D pseudo-NMR spectrum of an unknown biomarker. Correlation coefficients to the other signals in the median olive oil NMR spectrum are color-encoded: “driver peak” was at 5.907 ppm.

Figure S11. Box plots of a selection of statistically significant markers in the European sample set. Specifically, Saturated fatty acids (SFAs), Squalene and Total Phenols are depicted (vertical axis expressed in normalized intensity). SPA: Spain, GRE: Greece, ITA: Italy.

Figure S12. Box plots of a selection of statistically significant markers in the Greek sample set. Specifically, Oleocanthal, SFAs and Unsaturated fatty acids (UFAs) are depicted (vertical axis expressed in normalized intensity).

Table S1. Geographical origin of European samples.

ID	Country	Region	ID	Country	Region
13148	blend EU	blend EU	13649	ITA	ITA 100%
13150	blend EU	blend EU	13651	ITA	ITA 100%
13151	ITA	DOP Terra di Bari	13655	blend EU (SPA/GRE/IT)	blend EU
13153	SPA	SPA 100%	13656	ITA	DOP Terra di Bari
13157	SPA	SPA 100%	13660	blend EU	blend EU
13158	ITA	ITA 100%	13661	GRE	GRE 100%
13160	blend EU (SPA/GRE/IT)	blend EU	13662	ITA	ITA 100%
13161	SPA	SPA 100%	13667	ITA	ITA 100%
13164	SPA	SPA 100%	13670	GRE	Chania g.g.A.
13168	SPA	SPA 100%	13672	GRE	GRE 100%
13172	SPA	SPA 100%	13673	GRE	GRE 100%
13173	GRE	PDO Sitia/Kreta	13674	ITA	ITA 100%
13174	GRE	GRE 100%	13675	ITA	ITA 100%
13176	blend EU	blend EU	13677	GRE	PDO Sitia/Kreta
13178	blend EU	blend EU	13678	ITA	DOP Terra di Bari
13179	blend EU	blend EU	13680	SPA	SPA 100%
13180	blend EU (SPA/GRE/IT)	blend EU	13682	SPA	SPA 100%
13181	SPA	SPA 100%	13683	GRE	GRE 100%
13182	SPA	SPA 100%	13685	GRE	GRE 100%
13183	blend EU (SPA/GRE/IT)	blend EU	13686	GRE	GRE 100%
13184	SPA	SPA 100%	13687	blend EU (SPA/GRE/IT)	blend EU
13186	SPA	SPA 100%	13691	ITA	ITA 100%
13189	blend EU	blend EU	13692	blend EU (SPA/GRE/IT)	blend EU
13190	GRE	GRE 100%	13694	blend EU	blend EU
13191	GRE	Chania g.g.A.	13698	blend EU	blend EU
13192	ITA	DOP Terra di Bari	13699	SPA	SPA 100%
13193	blend EU (SPA/GRE/IT)	blend EU	13702	SPA	SPA 100%
13195	blend EU	blend EU	13703	SPA	SPA 100%
13196	blend EU (SPA/GRE/IT)	blend EU	13704	ITA	DOP Terra di Bari
13204	SPA	SPA 100%	13707	GRE	PDO Sitia/Kreta
13602	blend EU (SPA/GRE/IT)	blend EU	13708	ITA	DOP Terra di Bari
13606	GRE	GRE 100%	13710	GRE	PDO Sitia/Kreta
13607	SPA	SPA 100%	13711	SPA	SPA 100%
13609	blend EU	blend EU	13714	ITA	DOP Terra di Bari
13611	GRE	PDO Sitia/Kreta	13715	blend EU	blend EU
13614	GRE	GRE PDO	13722	blend EU	blend EU
13615	GRE	GRE 100%	13723	blend EU	blend EU
13616	SPA	SPA 100%	13726	SPA	blend EU
13619	GRE	PDO Vorios Mylo-potamos	13727	GRE	PDO Sitia/Kreta
13621	blend EU	blend EU	13728	ITA	DOP Terra di Bari
13622	blend EU	blend EU	13734	SPA	SPA 100%
13623	SPA	SPA 100%	13735	SPA	SPA 100%
13625	SPA	SPA 100%	13737	blend EU	blend EU
13627	GRE	PDO Vorios Mylo-potamos	13739	ITA	DOP Terra di Bari
13631	blend EU	blend EU	13740	SPA	SPA 100%

ID	Country	Region	ID	Country	Region
13632	blend EU	blend EU	13743	GRE	GRE 100%
13633	GRE	PDO Vorios Mylo-potamos	13746	blend EU	blend EU
13635	ITA	ITA 100%	13747	GRE	GRE 100%
13638	SPA	SPA 100%	13748	GRE	GRE 100%
13641	GRE	PDO Sitia/Kreta	13750	GRE	PDO Sitia/Kreta
13642	GRE	GRE 100%	13751	SPA	SPA 100%
13645	blend EU	blend EU	13752	ITA	DOP Terra di Bari
13646	blend EU	blend EU	13753	SPA	SPA 100%
13647	GRE	GRE 100%	13755	blend EU (SPA/GRE/IT)	blend EU
13648	SPA	SPA 100%	13756	blend EU (SPA/GRE/IT)	blend EU

*ITA: Italy, SPA: Spain and GRE: Greece

Table S2. Geographical and botanical origin of Greek samples.

ID	Region	Subregion	Variety
LSCR91	North Aegean-Crete	North Aegean 90%-Crete 10%	Koroneiki
LSCR82	North Aegean-Crete	North Aegean 80%-Crete 20%	Koroneiki
LSCR73	North Aegean-Crete	North Aegean 70%-Crete 30%	Koroneiki
LSCR64	North Aegean-Crete	North Aegean 60%-Crete 40%	Koroneiki
LSCR55	North Aegean-Crete	North Aegean 50%-Crete 50%	Koroneiki
CRLS64	North Aegean-Crete	North Aegean 40%-Crete 60%	Koroneiki
CRLS73	North Aegean-Crete	North Aegean 30%-Crete 70%	Koroneiki
CRLS82	North Aegean-Crete	North Aegean 20%-Crete 80%	Koroneiki
CRLS91	North Aegean-Crete	North Aegean 10%-Crete 90%	Koroneiki
LSPL91	North Aegean-Peloponnese	North Aegean 90%-Peloponnese 10%	Koroneiki
LSPL82	North Aegean-Peloponnese	North Aegean 80%-Peloponnese 20%	Koroneiki
LSPL73	North Aegean-Peloponnese	North Aegean 80%-Peloponnese 20%	Koroneiki
LSPL64	North Aegean-Peloponnese	North Aegean 80%-Peloponnese 20%	Koroneiki
LSPL55	North Aegean-Peloponnese	North Aegean 80%-Peloponnese 20%	Koroneiki
PLLS64	North Aegean-Peloponnese	North Aegean 80%-Peloponnese 20%	Koroneiki
PLLS73	North Aegean-Peloponnese	North Aegean 80%-Peloponnese 20%	Koroneiki
PLLS82	North Aegean-Peloponnese	North Aegean 80%-Peloponnese 20%	Koroneiki
PLLS91	North Aegean-Peloponnese	North Aegean 80%-Peloponnese 20%	Koroneiki
KRAD91	North Aegean	Lesvos	Koroneiki 90%-Adramitiani 10%
KRAD82	North Aegean	Lesvos	Koroneiki 80%-Adramitiani 20%
KRAD73	North Aegean	Lesvos	Koroneiki 70%-Adramitiani 30%
KRAD64	North Aegean	Lesvos	Koroneiki 60%-Adramitiani 40%
ADKR55	North Aegean	Lesvos	Koroneiki 50%-Adramitiani 50%
ADKR64	North Aegean	Lesvos	Koroneiki 40%-Adramitiani 60%
ADKR73	North Aegean	Lesvos	Koroneiki 30%-Adramitiani 70%
ADKR82	North Aegean	Lesvos	Koroneiki 20%-Adramitiani 80%
ADKR91	North Aegean	Lesvos	Koroneiki 10%-Adramitiani 90%
CRPL91	Crete-Peloponnese	Crete 90%-Peloponnese 10%	Koroneiki
CRPL82	Crete-Peloponnese	Crete 80%-Peloponnese 20%	Koroneiki
CRPL73	Crete-Peloponnese	Crete 70%-Peloponnese 30%	Koroneiki
CRPL64	Crete-Peloponnese	Crete 60%-Peloponnese 40%	Koroneiki
PLCR55	Crete-Peloponnese	Crete 50%-Peloponnese 50%	Koroneiki
PLCR64	Crete-Peloponnese	Crete 40%-Peloponnese 60%	Koroneiki

ID	Region	Subregion	Variety
PLCR73	Crete-Peloponnese	Crete 30%-Peloponnese 70%	Koroneiki
PLCR82	Crete-Peloponnese	Crete 20%-Peloponnese 80%	Koroneiki
PLCR91	Crete-Peloponnese	Crete 10%-Peloponnese 90%	Koroneiki
KLKR91	North Aegean	Lesvos	Kolovi 90%-Koroneiki 10%
KLKR82	North Aegean	Lesvos	Kolovi 80%-Koroneiki 20%
KLKR73	North Aegean	Lesvos	Kolovi 70%-Koroneiki 30%
KLKR64	North Aegean	Lesvos	Kolovi 60%-Koroneiki 40%
KRKL55	North Aegean	Lesvos	Kolovi 50%-Koroneiki 50%
KRKL64	North Aegean	Lesvos	Kolovi 40%-Koroneiki 60%
KRKL73	North Aegean	Lesvos	Kolovi 30%-Koroneiki 70%
KRKL82	North Aegean	Lesvos	Kolovi 20%-Koroneiki 80%
KRKL91	North Aegean	Lesvos	Kolovi 10%-Koroneiki 90%
KLAD91	North Aegean	Lesvos	Kolovi 90%-Adramitiani 10%
KLAD82	North Aegean	Lesvos	Kolovi 80%-Adramitiani 20%
KLAD73	North Aegean	Lesvos	Kolovi 70%-Adramitiani 30%
KLAD64	North Aegean	Lesvos	Kolovi 60%-Adramitiani 40%
ADKL55	North Aegean	Lesvos	Kolovi 50%-Adramitiani 50%
ADKL64	North Aegean	Lesvos	Kolovi 40%-Adramitiani 60%
ADKL73	North Aegean	Lesvos	Kolovi 30%-Adramitiani 70%
ADKL82	North Aegean	Lesvos	Kolovi 20%-Adramitiani 80%
ADKL91	North Aegean	Lesvos	Kolovi 10%-Adramitiani 90%
KLAO1	Crete	Lasithi	Koroneiki
KLAO2	Crete	Lasithi	Koroneiki
KLAO3	Crete	Lasithi	Koroneiki
KLAO4	Crete	Lasithi	Koroneiki
KLAO5	Crete	Lasithi	Koroneiki
KLAO6	Crete	Lasithi	Koroneiki
KLAO7	Crete	Lasithi	Koroneiki
KLO1	North Aegean	Lesvos	Koroneiki
KLLO2	North Aegean	Lesvos	Kolovi
KLLO3	North Aegean	Lesvos	Kolovi
KLLO4	North Aegean	Lesvos	Kolovi
KLLO5	North Aegean	Lesvos	Kolovi
KLO6	North Aegean	Lesvos	Koroneiki
KLLO7	North Aegean	Lesvos	Kolovi
KLO8	North Aegean	Lesvos	Koroneiki
KLO9	North Aegean	Lesvos	Koroneiki
ALO10	North Aegean	Lesvos	Adramitiani
KLLO11	North Aegean	Lesvos	Kolovi
KLLO12	North Aegean	Lesvos	Kolovi
KLLO13	North Aegean	Lesvos	Kolovi
KLO14	North Aegean	Lesvos	Koroneiki
KLLO15	North Aegean	Lesvos	Kolovi
KLO16	North Aegean	Lesvos	Koroneiki
KRO1	Crete	Rethymno	Koroneiki
KRO2	Crete	Rethymno	Koroneiki
KRO3	Crete	Rethymno	Koroneiki
KRO4	Crete	Rethymno	Koroneiki

ID	Region	Subregion	Variety
KRO5	Crete	Rethymno	Koroneiki
KRO6	Crete	Rethymno	Koroneiki
KRO7	Crete	Rethymno	Koroneiki
KRO8	Crete	Rethymno	Koroneiki
KHO1	Crete	Herakleio	Koroneiki
KHO2	Crete	Herakleio	Koroneiki
KHO3	Crete	Herakleio	Koroneiki
KHO4	Crete	Herakleio	Koroneiki
KHO5	Crete	Herakleio	Koroneiki
KHO6	Crete	Herakleio	Koroneiki
KHO7	Crete	Herakleio	Koroneiki
KHO8	Crete	Herakleio	Koroneiki
KHO9	Crete	Herakleio	Koroneiki
KHO10	Crete	Herakleio	Koroneiki
KHO11	Crete	Herakleio	Koroneiki
KHO12	Crete	Herakleio	Koroneiki
KRCH1	Crete	Chania	Koroneiki
KRCH2	Crete	Chania	Koroneiki
KRCH3	Crete	Chania	Koroneiki
KRCH4	Crete	Chania	Koroneiki
KRCH5	Crete	Chania	Koroneiki
KRCH6	Crete	Chania	Koroneiki
KRCH7	Crete	Chania	Koroneiki
KRCH8	Crete	Chania	Koroneiki
KRCH9	Crete	Chania	Koroneiki
KMO1	Peloponnese	Messinia	Koroneiki
KMO2	Peloponnese	Messinia	Koroneiki
KMO3	Peloponnese	Messinia	Koroneiki
KMO4	Peloponnese	Messinia	Koroneiki
KMO5	Peloponnese	Messinia	Koroneiki
KMO6	Peloponnese	Messinia	Koroneiki
KMO7	Peloponnese	Messinia	Koroneiki
KMO8	Peloponnese	Messinia	Koroneiki
KMO9	Peloponnese	Messinia	Koroneiki
KMO10	Peloponnese	Messinia	Koroneiki
KMO11	Peloponnese	Messinia	Koroneiki
KMO12	Peloponnese	Messinia	Koroneiki
KLLK2	Peloponnese	Lakonia	Unknown
MXLK1	Peloponnese	Lakonia	Unknown
KULK3	Peloponnese	Lakonia	Unknown
KRLK4	Peloponnese	Lakonia	Koroneiki
AKZO1	Ionian Islands	Zakynthos	Koroneiki
AMAO1	Peloponnese	Argolida	Megaritiki
AM-NAO1	Peloponnese	Argolida	Manaki
AKFO1	Peloponnese	Messinia	Koroneiki
AKZO2	Ionian Islands	Zakynthos	Koroneiki
ANZO1	Ionian Islands	Zakynthos	Ntopia

ID	Region	Subregion	Variety
AKKO1	Peloponnese	Messinia	Koroneiki
AKKO2	Peloponnese	Messinia	Koroneiki
ATKO1	Ionian Islands	Kefalonia	Thiaki

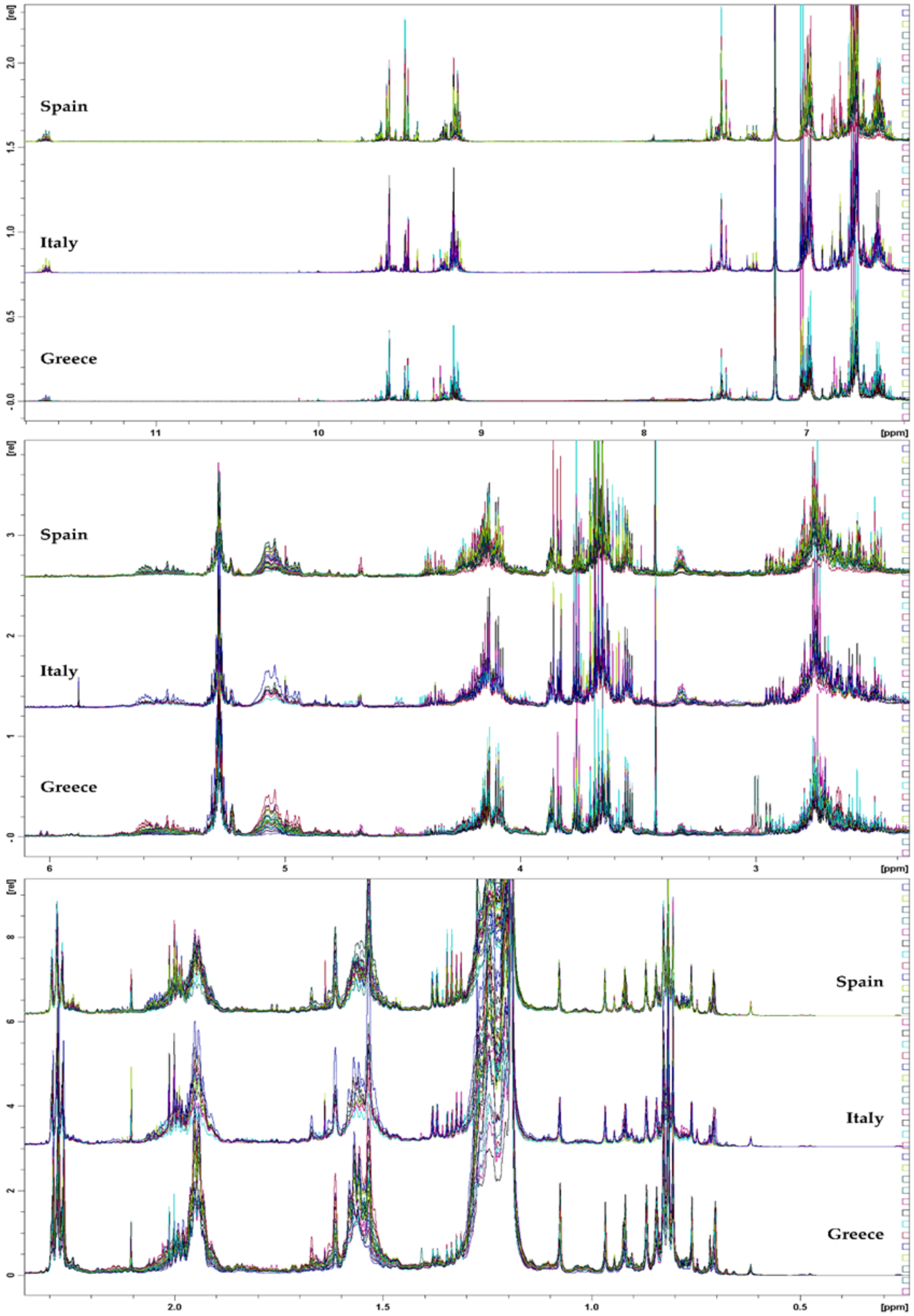

Figure S1. Multiple display of spectra from Spain, Italy and Greece. Quantitative variations are mainly observed across the field.

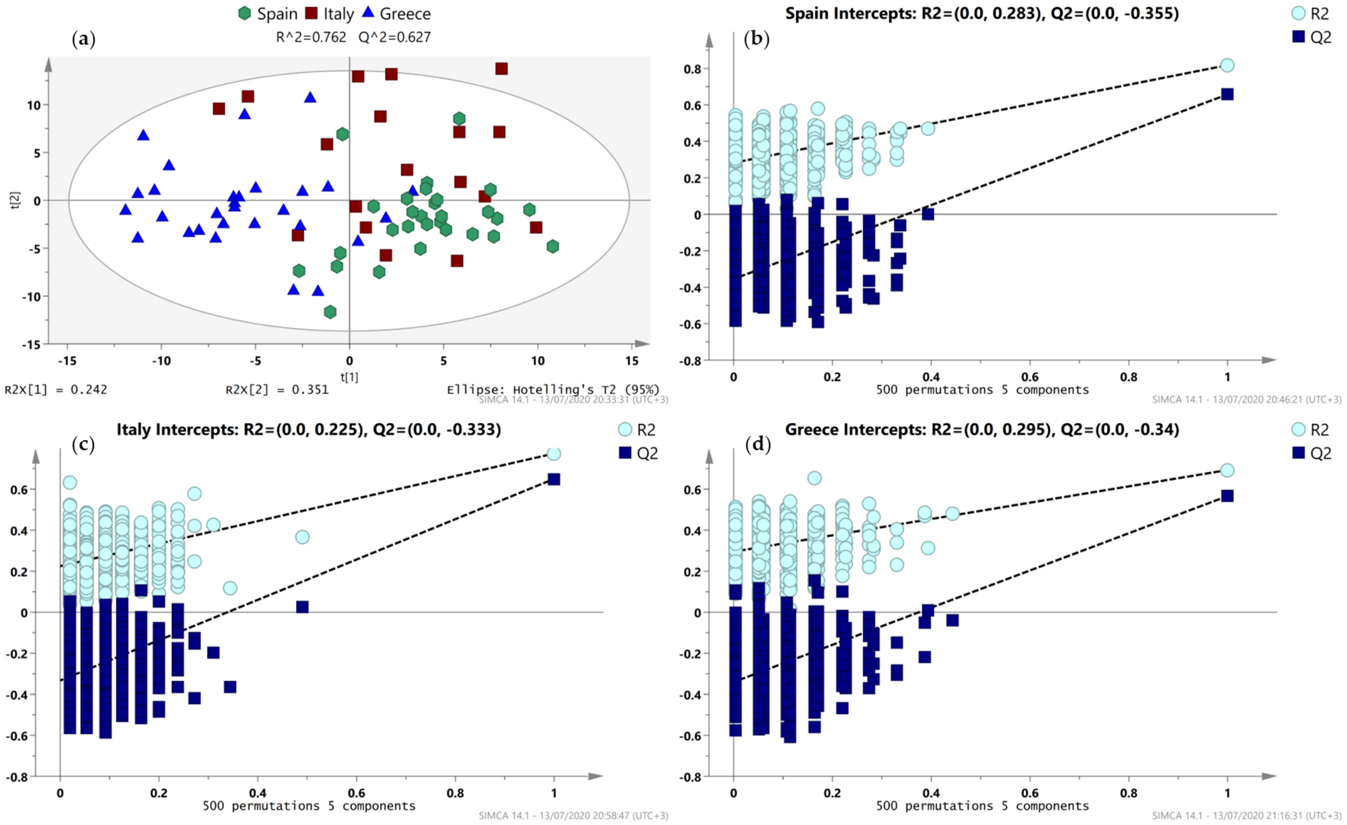

Figure S2. (**a**) PLS-DA scores scatter plot of the European sample set with a clear distinction of samples from Greece; (**b**) Permutation test with 500 permutations performed at the presented PLS-DA model with samples from Spain; (**c**) Respective permutation test with samples from Italy; (**d**) Respective permutation test with samples from Greece.

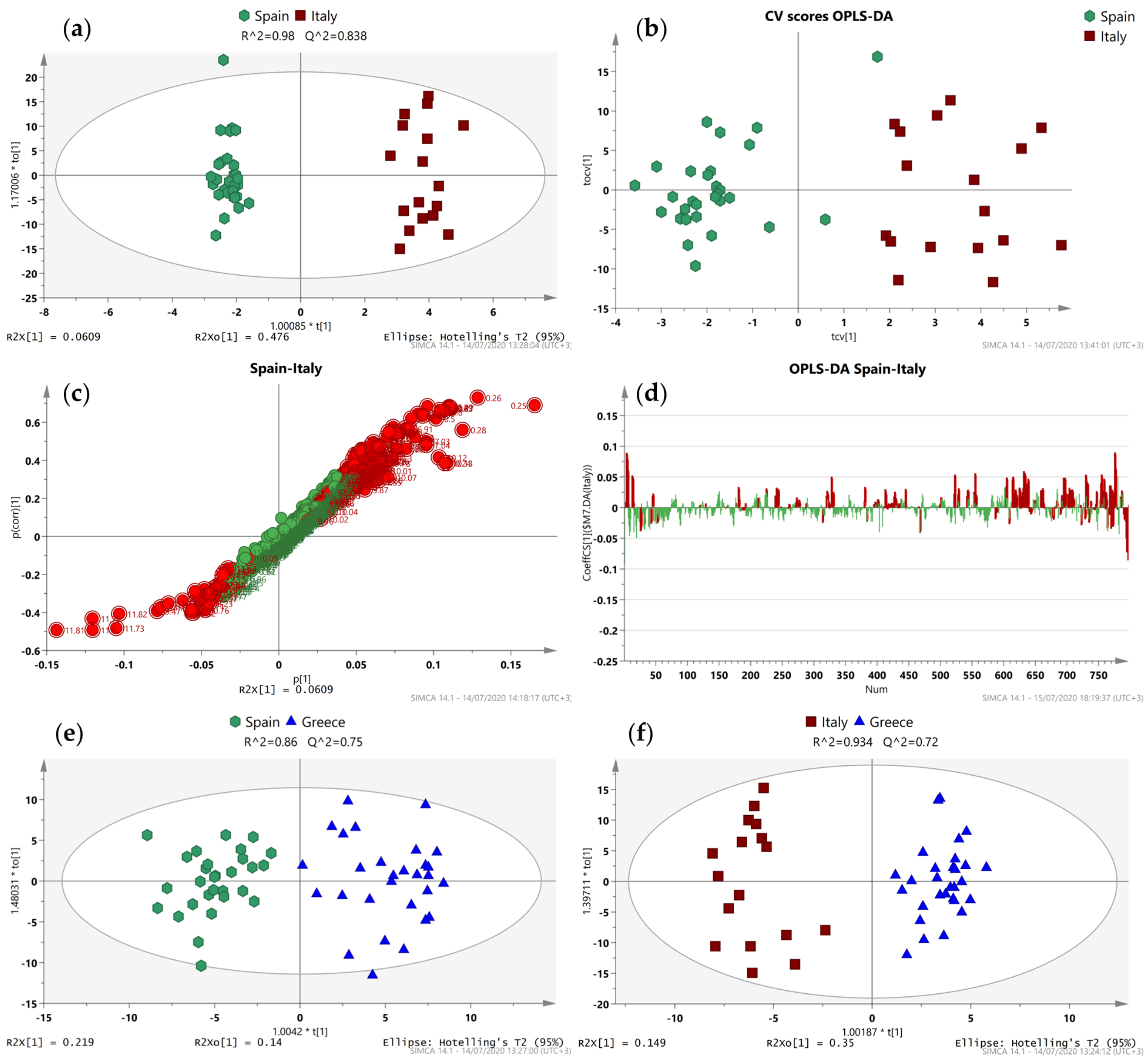

Figure S3. (**a**) OPLS-DA scores scatter plot with samples from Spain vs Italy; (**b**) Indicative CV scores scatter plot from the OPLS-DA model of Spain vs Italy; (**c**) Indicative S-plot from the OPLS-DA model of Spain vs Italy; (**d**) Indicative Coefficients plot from OPLS-DA model of Spain vs Italy; (**e**) OPLS-DA scores scatter plot with samples from Spain vs Greece; (**f**) OPLS-DA scores scatter plot with samples from Italy vs Greece.

Table S3. Statistically significant markers extracted from the respective OPLS-DA models of European oils. Variable ID (ppm), multiplicity/functional group, VIP and p(corr) values along with the respective class are presented.

No	Var ID	Multiplicity / Functional group	Proposed Compound	VIP	p(corr)	Class
**OPLS-DA / Italy vs Greece**						
1	0.48	s / -CH_3 (H-18)	Gramisterol/citrostadienol/Δ7-avenasterol/Δ7-campesterol	1.57376	0.70169	Greece
2	0.71	s / -CH_3	Triterpene	1.85219	0.75826	Greece
3	0.76	s / -CH_3	Triterpene	1.73687	0.76891	Greece
4	0.8	t / -CH_3	Saturated, oleic and/or ω-7 acyl groups	1.63301	0.73946	Greece

No	Var ID	Multiplicity / Functional group	Proposed Compound	VIP	p(corr)	Class
5	0.84	overlapping signal / $-CH_3$	Triterpene /Linoleic acyl groups	1.96608	0.80001	Greece
6	0.85	s / $-CH_3$	Triterpene	1.58902	0.71504	Greece
7	0.87	s / $-CH_3$	Triterpene	1.78751	0.75057	Greece
8	0.92	s / $-CH_3$	Triterpene	1.88684	0.79808	Greece
9	0.97	s / $-CH_3$	Triterpene	1.76052	0.75217	Greece
10	1.08	s / $-CH_3$	Triterpene	1.75453	0.73571	Greece
11	4.36	-	Unknown	1.30686	-0.73187	Italy
12	5.22	m / olefinic H (H-12)	Triterpene	2.05517	0.74127	Greece
**OPLS-DA / Spain vs Italy**						
1	0.26	d / $-CH_2-$ (exo, H-19)	Cycloartenol/cyclobranol/24-methylenecycloartanol	2.69135	0.73180	Italy
**OPLS-DA / Spain vs Greece**						
1	0.48	s / -CH3 (H-18)	Gramisterol/citrostadienol/Δ7-avenasterol/Δ7-campesterol	1.57158	0.81217	Greece
2	0.66	-	Unknown	1.68377	0.84102	Greece
3	0.67	-	Unknown	1.41605	0.79866	Greece
4	0.68	-	Unknown	1.85150	0.86148	Greece
5	0.69	-	Unknown	1.50375	0.79145	Greece
6	0.71	s / -CH3	Triterpene	1.51902	0.82189	Greece
7	0.72	s / -CH3	Triterpene	1.90038	0.86625	Greece
8	0.73	s / -CH3	Triterpene	1.60446	0.85985	Greece
9	0.76	s / -CH3	Triterpene	1.47770	0.87314	Greece
10	0.79	s / -CH3	Triterpene	1.28447	0.81407	Greece
11	0.82	t / -CH3	Saturated, oleic and/or ω-7 acyl groups	1.78416	0.83707	Greece
12	0.84	overlapping signal / -CH3	Triterpene /Linoleic acyl groups	1.79873	0.91611	Greece
13	0.85	s / -CH3	Triterpene	1.36544	0.79072	Greece
14	0.87	s / -CH3	Triterpene	1.56433	0.84776	Greece
15	0.88	-	Unknown	1.49806	0.83271	Greece
16	0.89	-	Unknown	1.60573	0.88304	Greece
17	0.9	-	Unknown	1.39417	0.85199	Greece
18	0.91	-	Unknown	1.40856	0.85011	Greece
19	0.92	s / -CH3	Triterpene	1.67697	0.89908	Greece
20	0.93	s / -CH3	Triterpene	1.60753	0.85442	Greece
21	0.95	s / -CH3	Triterpene	1.12868	0.72360	Greece
22	0.97	s / -CH3	Triterpene	1.54112	0.87273	Greece
23	1	-	Unknown	1.26887	0.76788	Greece
24	1.01	-	Unknown	1.39883	0.83934	Greece
25	1.02	-	Unknown	1.17752	0.76622	Greece
26	1.03	-	Unknown	1.50154	0.85059	Greece
27	1.04	-	Unknown	1.22220	0.77273	Greece
28	1.08	s / -CH3	Triterpene	1.58526	0.85248	Greece
29	1.1	-	Unknown	1.29374	0.80272	Greece
30	1.11	-	Unknown	1.29219	0.79664	Greece
31	1.12	-	Unknown	1.17649	0.73321	Greece
32	1.14	-	Unknown	1.24096	0.75478	Greece

No	Var ID	Multiplicity / Functional group	Proposed Compound	VIP	p(corr)	Class
33	1.19	m / (-CH2-)n	Saturated fatty acid (SFA) chains	1.40242	0.82194	Greece
34	1.24	m / (-CH2-)n	Unsaturated fatty acid (UFA) chains	1.77041	0.83468	Greece
35	1.4	-	Unknown	1.33746	0.79922	Greece
36	1.46	-	Unknown	1.23594	0.70613	Greece
37	1.48	-	Unknown	1.22365	0.72721	Greece
38	1.57	m / -OCO-CH2-CH2-	Triacylglycerols (TAGs) - Acyl groups	1.72289	0.86111	Greece
39	1.85	-	Unknown	1.18790	0.72523	Greece
40	1.95	m / -CH2-CH=CH-	TAGs - Acyl groups	1.74848	0.83021	Greece
41	2.28	m / -OCO-CH2-	TAGs - Acyl groups	1.82255	0.86162	Greece
42	2.94	d (J=9.6 Hz) / -CH(OH)-	Triterpene	1.12645	0.71403	Greece
43	3.69	-	Unknown	1.54239	-0.75428	Spain
44	3.86	s / -OCH3 (H-7')	Acetoxypinoresinol	1.15493	0.73396	Greece
45	3.87	-	Unknown	1.08121	0.73010	Greece
46	5.22	m / olefinic H (H-12)	Triterpene	2.12264	0.90783	Greece
47	5.28	m / -CH=CH-	TAGs - Acyl groups	1.94420	0.84567	Greece
48	5.39	-	Unknown	1.44541	0.78876	Greece
49	9.41	-CH=O	Unknown	1.56219	-0.71737	Spain
50	9.64	-CH=O	Unknown	1.66357	-0.70737	Spain
51	9.65	-CH=O	Unknown	1.70797	-0.71366	Spain
52	9.74	-CH=O	Unknown	1.67958	-0.74730	Spain
53	11.7	-OH	Unknown	1.83856	-0.73099	Spain
54	11.72	-OH	Unknown	2.02786	-0.71144	Spain

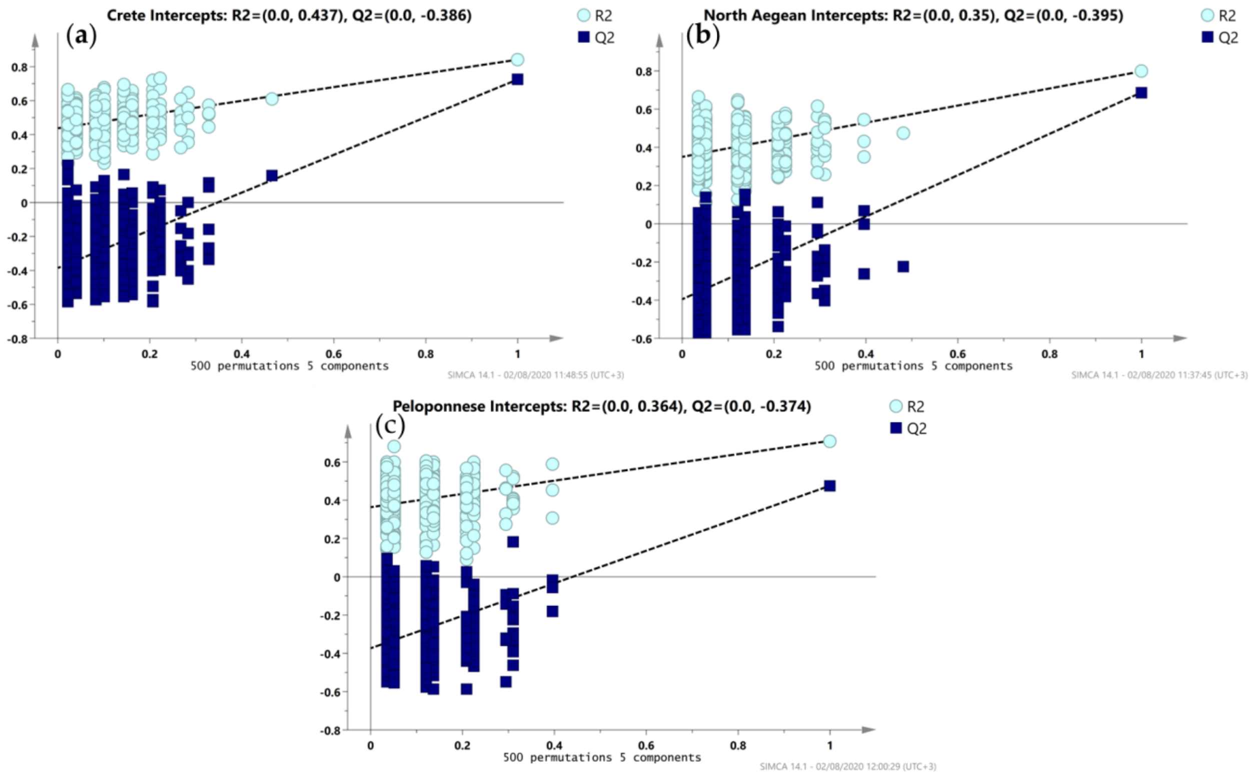

Figure S4. (**a**) Permutation test with 500 permutations performed for PLS-DA model in Figure 2 with samples from Crete; (**b**) Respective permutation test with samples from North Aegean; (**c**) Respective permutation test with samples from Peloponnese.

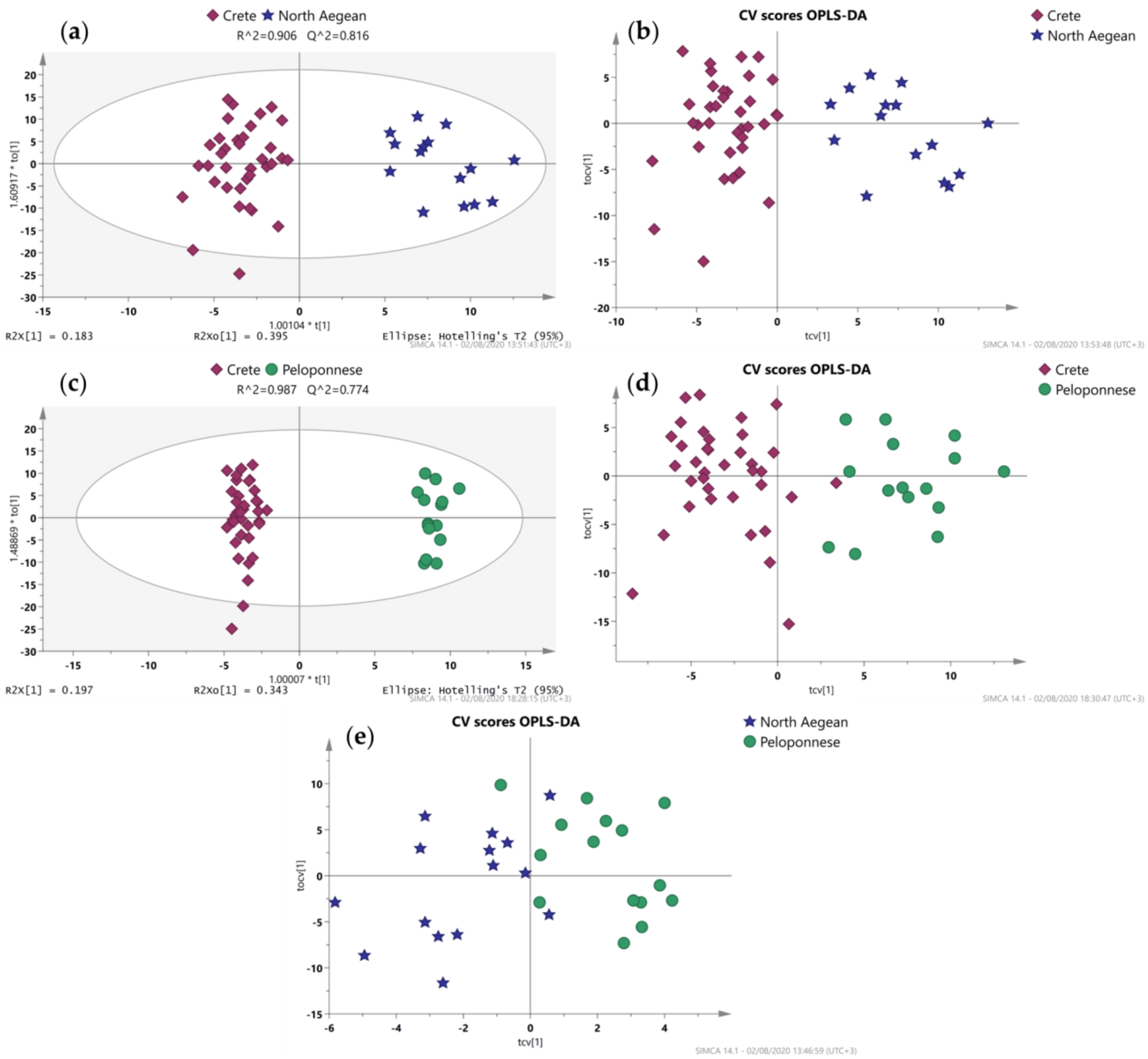

Figure S5. (**a**) OPLS-DA scores scatter plot with samples from Crete vs North Aegean; (**b**) CV scores scatter plot from the OPLS-DA model of Crete vs North Aegean; (**c**) OPLS-DA scores scatter plot with samples from Crete vs Peloponnese; (**d**) CV scores scatter plot from the OPLS-DA model of Crete vs Peloponnese; (**e**) CV scores scatter plot from the OPLS-DA model of North Aegean vs Peloponnese.

Table S4. Statistically significant markers extracted from the respective OPLS-DA models of Greek oils. Variable ID (ppm), multiplicity/functional group, VIP and p(corr) values along with the respective class are presented.

No	Var ID	Multiplicity / Functional group	Proposed Compound	VIP	p(corr)	Class
**OPLS-DA / North Aegean vs Peloponnese**						
1	5.89	m	Unknown	2.51707	-0.790794	North Aegean
2	5.9	m	Unknown	2.82985	-0.823117	North Aegean
3	5.91	m	Unknown	2.39897	-0.742319	North Aegean
4	5.92	m	Unknown	2.15888	-0.754852	North Aegean
5	6.05	-	Unknown	2.25229	-0.771316	North Aegean
6	6.4	m	Unknown	2.4761	-0.734855	North Aegean
7	6.42	m	Unknown	2.80879	-0.791951	North Aegean
8	6.43	m	Unknown	2.293	-0.775474	North Aegean

No	Var ID	Multiplicity / Functional group	Proposed Compound	VIP	p(corr)	Class
9	6.44	m	Unknown	2.20413	-0.786371	North Aegean
10	7.43	-CH= (aromatic)	Unknown	2.50246	-0.81262	North Aegean
**OPLS-DA / Crete vs North Aegean**						
1	0.72	s / -CH3	Triterpene	1.50238	-0.704769	Crete
2	2.93	ddd (*J*=18.4/8.7/1.2 Hz) / -$\underline{CH_2}$-CH=O (H-4a)	Oleocanthal	1.42929	-0.719699	Crete
3	2.94	d (*J*=9.6 Hz) / -$\underline{CH}$(OH)-)	Triterpene	1.22126	-0.743346	Crete
4	3.15	dd (*J*=4.5/11.3 Hz) / -$\underline{CH}$(OH)-)	Triterpene	1.72675	-0.848694	Crete
5	3.17	dd (*J*=4.5/11.3 Hz) / -$\underline{CH}$(OH)-)	Triterpene	1.64105	-0.785879	Crete
6	5.23	m / olefinic H (H-12)	Triterpene	1.18199	-0.730016	Crete
7	9.22	-	Unknown	1.5768	-0.749416	Crete
8	9.7	-	Unknown	1.26164	-0.708038	Crete
**OPLS-DA / Crete vs Peloponnese**						
1	2	d (*J*=7.1 Hz) / -CH_3 (H-10)	Oleacein/Oleocanthal	1.48243	-0.708224	Crete
2	2.01	d (*J*=7.1 Hz) / -CH_3 (H-10)	Oleocanthal	1.48572	-0.706204	Crete
3	2.93	ddd (*J*=18.4/8.7/1.2 Hz) / -$\underline{CH_2}$-CH=O (H-4a)	Oleocanthal	1.34374	-0.712692	Crete
4	3.53	dd (*J*=5.8/11.5 Hz) / -$\underline{CH_2}$–OCO- (H-3'a)	Glycerol in 1-MAG	1.10222	-0.701298	Crete
5	6.98	d (*J*=8.5 Hz) / aromatic H (H-4/H-8 or H-4'/H-8')	Tyrosol & derivatives	1.68536	-0.733814	Crete
6	6.99	d (*J*=8.5 Hz) / aromatic H (H-4/H-8 or H-4'/H-8')	Tyrosol & derivatives	1.59958	-0.726112	Crete
7	9.2	-CH=O	Unknown	1.35654	-0.712554	Crete
8	9.7	-CH=O	Unknown	1.39785	-0.776816	Crete
**OPLS-DA / Koroneiki vs Kolovi**						
1	2.71	t / (=HC-CH2-CH=)	Linoleic acyl groups	1.91179	0.708193	Kolovi
2	6.05	-	Unknown	1.73997	0.846117	Kolovi
3	6.11	-	Unknown	1.4621	0.738442	Kolovi
4	6.13	-	Unknown	1.7987	0.869632	Kolovi
5	7.43	-CH= (aromatic)	Unknown	1.49286	0.715732	Kolovi

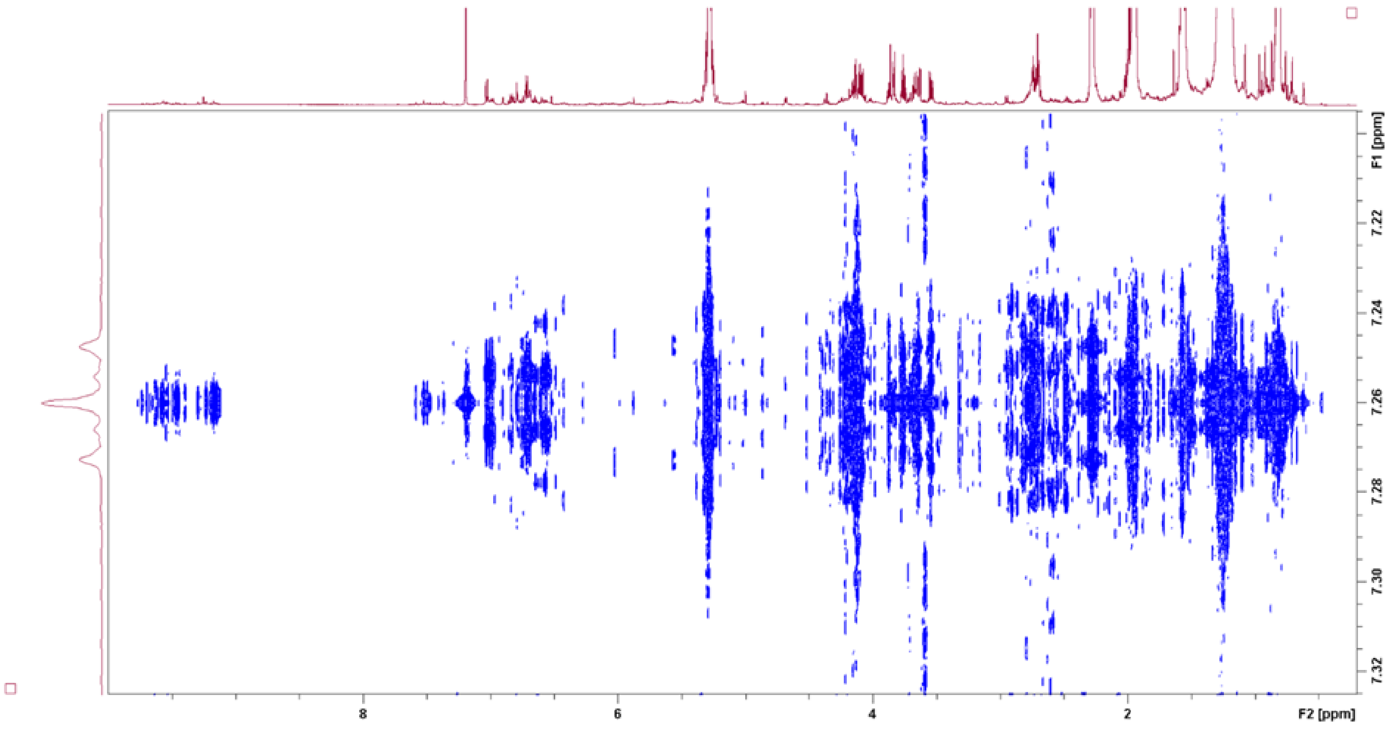

Figure S6. Representative JRES spectrum of a Greek EVOO.

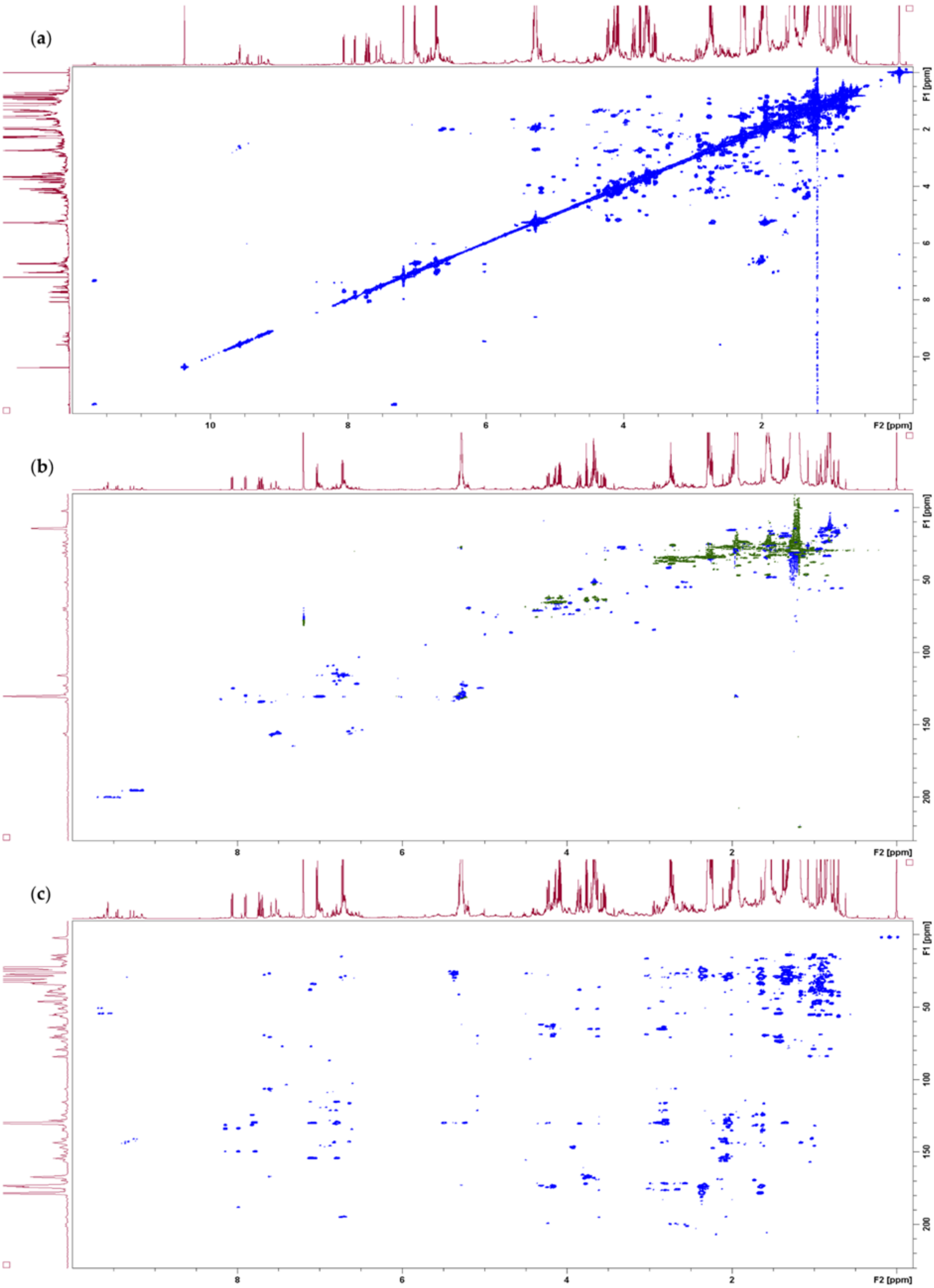

Figure S7. Representative 2D spectra of a Greek EVOO. (**a**) COSY; (**b**) HSQC-DEPT; (**c**) HMBC.

Table S5. ^{1}H NMR chemical shifts and assignments for EVOOs' metabolites identified. Multiplicity (*J* in Hz) and functional group are also presented.

δ ^{1}H	Multiplicity/Functional group	Proposed Compound
0.26	d / -CH2- (exo, H-19)	Cycloartenol/cyclobranol/24-methylenecycloartanol
0.48	s / -CH3 (H-18)	Gramisterol/citrostadienol/Δ7-avenasterol/Δ7-campesterol
0.49	d / -CH2- (endo, H-19)	Cycloartenol/cyclobranol/24-methylenecycloartanol
0.61	s / -CH$_3$ (H-18)	β-sitosterol/Δ5-avenasterol/Δ5-campesterol
0.71	s / -CH$_3$	Triterpene
0.72	s / -CH$_3$	Triterpene
0.73	s / -CH$_3$	Triterpene
0.76	s / -CH$_3$	Triterpene
0.79	s / -CH$_3$	Triterpene
0.81-0.83	t / -CH$_3$	Saturated, oleic and/or ω-7 acyl groups
0.84	overlapping signal / -CH$_3$	Triterpene /Linoleic acyl groups
0.85	s / -CH$_3$	Triterpene
0.87	s / -CH$_3$	Triterpene
0.92	s / -CH$_3$	Triterpene
0.93	s / -CH$_3$	Triterpene
0.95	s / -CH$_3$	Triterpene
0.96	s / -CH$_3$	Triterpene
0.97	s / -CH$_3$	Triterpene
1.08	s / -CH$_3$	Triterpene
1.19	m / (-CH$_2$-)$_n$	SFA chains
1.24	m / (-CH$_2$-)$_n$	UFA chains
1.31-1.38	d (*J*=6.7 Hz) / -CH$_3$ (H-10)	Elenolic acid/elenolic acid methyl-or ethylester/Monoaldehydic form of ligstroside aglycon (MFLA)/monoaldehydic form of oleuropein aglycon (MFOA) (5S,8R,9S)
1.46-1.52	d (*J*=6.7 Hz) / -CH$_3$ (H-10)	Elenolic acid/elenolic acid methyl-or ethylester/MFLA/MFOA (5S,8S,9S)
1.53	s / -CH$_3$ (H-8/H-9)	Squalene
1.56	m / -OCO-CH$_2$-CH$_2$-	TAGs - Acyl groups
1.57	m / -OCO-CH$_2$-CH$_2$-	TAGs - Acyl groups
1.61	s / -CH$_3$ (H-10)	Squalene
1.64	s / CH$_3$-COO- (H-9)	Acetoxypinoresinol
1.95	m / -CH$_2$-CH=CH-	TAGs - Acyl groups
1.99	d (*J*=7.1 Hz) / -CH$_3$ (H-10)	Oleacein
2.00	d (*J*=7.1 Hz) / -CH$_3$ (H-10)	Oleacein/Oleocanthal
2.01	d (*J*=7.1 Hz) / -CH$_3$ (H-10)	Oleocanthal
2.26-2.31	m / -OCO-CH$_2$-	TAGs - Acyl groups
2.71	t / =HC-CH$_2$-CH=	Linoleic acyl groups
2.84-2.89	ddd (*J*=18.4/8.2/1.1 Hz) / -CH$_2$-CH=O (H-4a)	Oleacein
2.88-2.93	ddd (*J*=18.4/8.7/1.2 Hz) / -CH$_2$-CH=O (H-4a)	Oleocanthal
2.94	d (*J*=9.6 Hz) / -CH(OH)-	Triterpene

3.15	dd (*J*=4.5/11.3 Hz) / -CH(OH)-	Triterpene
3.52-3.56	dd (*J*=5.8/11.5 Hz) / -CH_2–OCO- (H-3'a)	Glycerol in 1-MAG
3.65	s / -OCH_3 (H-12)	MFLA (5S,8R,9S)
3.68	s / -OCH_3 (H-12)	MFOA (5S,8R,9S)
3.83	s / -OCH_3 (H-7'')	Acetoxypinoresinol
3.86	s / -OCH_3 (H-7')	Acetoxypinoresinol
4.08	dd (*J*=6.2/11.7 Hz) / -CH_2–OCO– (H-1'a/H-3'a)	Glycerol in sn-1,3 DAG
4.09	dd (*J*=6.1/11.7 Hz) / -CH_2–OCO– (H-1'a/H-3'a)	Glycerol in TAG
4.14	dd (*J*=4.5/11.7 Hz) / -CH_2–OCO– (H-1'b/H-3'b)	Glycerol in sn-1,3 DAG
4.25	dd / -CH_2–OCO– (H-1'a/H-1'b)	Glycerol in TAG
4.26	dd / -CH_2–OCO– (H-1'b)	Glycerol in sn-1,2 DAG
4.68	d (*J*=4.9 Hz) / tetrahydrofuranic H (H-6)	Acetoxypinoresinol
5.00	s / tetrahydrofuranic H (H-2)	Acetoxypinoresinol
5.22-5.23	m / olefinic H (H-12)	Triterpene
5.24-5.33	m / -CH=CH-	TAGs - Acyl groups
6.65	d (*J*=1.9 Hz) / aromatic H (H-4 or H-4')	Hydroxytyrosol & derivatives
6.69-6.74	d (*J*=8.0 Hz) / aromatic H (H-7 or H-7')	Hydroxytyrosol & derivatives
6.69-6.74	d (*J*=8.5 Hz) / aromatic H (H-5/H-7 or H-5'/H7')	Tyrosol & derivatives
6.96-7.05	d (*J*=8.5 Hz) / aromatic H (H-4/H-8 or H-4'/H8')	Tyrosol & derivatives
7.49	brs / -O-CH= (H-3)	MFLA (5S,8R,9S)
7.52	brs / -O-CH= (H-3)	MFOA (5S,8R,9S)
7.55	brs / -O-CH= (H-3)	MFLA (5S,8S,9S)
7.58	brs / -O-CH= (H-3)	MFOA (5S,8S,9S)
9.14	d (*J*=1.9 Hz) / -CH=O (H-1)	Oleacein
9.17	d (*J*=1.9 Hz) / -CH=O (H-1)	Oleocanthal
9.45	brd (*J*=1.8 Hz) / -CH=O (H-1)	MFLA (5S,8R,9S)
9.48	brd (*J*=1.8 Hz) / -CH=O (H-1)	MFOA (5S,8R,9S)
9.50	brd (*J*=1.8 Hz) / -CH=O (H-1)	MFLA (5S,8S,9S)
9.53	brd (*J*=1.8 Hz) / -CH=O (H-1)	MFOA (5S,8S,9S)
9.57	brs / -CH=O (H-3)	Oleocanthal
9.58	brs / -CH=O (H-3)	Olaecein

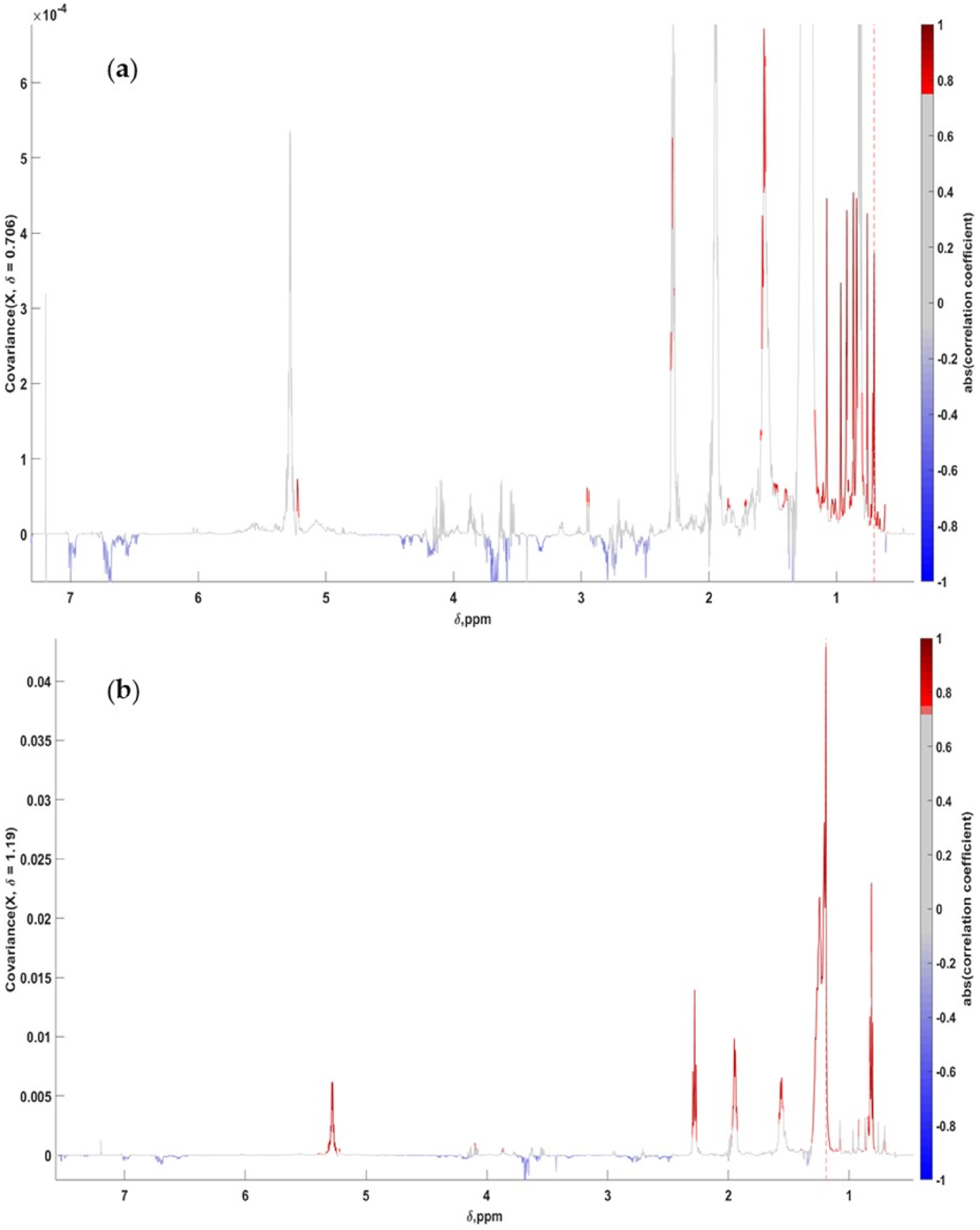

Figure S8. STOCSY 1D pseudo-NMR spectra. Correlation coefficients to the other signals in the median EVOO NMR spectrum are color-encoded. (**a**) Triterpenes: "driver peak" was at 0.706 ppm; (**b**) Fatty acids: "driver peak" was at 1.190 ppm.

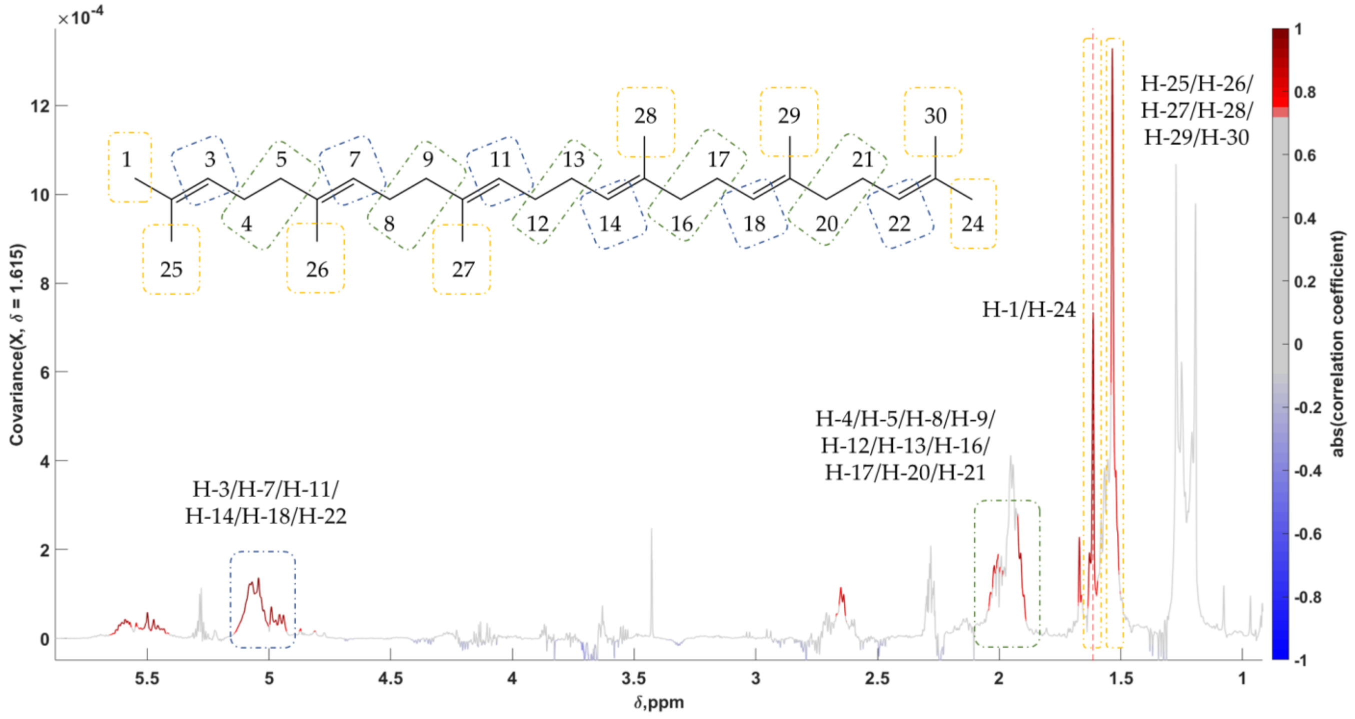

Figure S9. STOCSY 1D pseudo-NMR spectrum of squalene. Correlation coefficients to the other signals in the median EVOO NMR spectrum are color-encoded: “driver peak” was at 1.615 ppm.

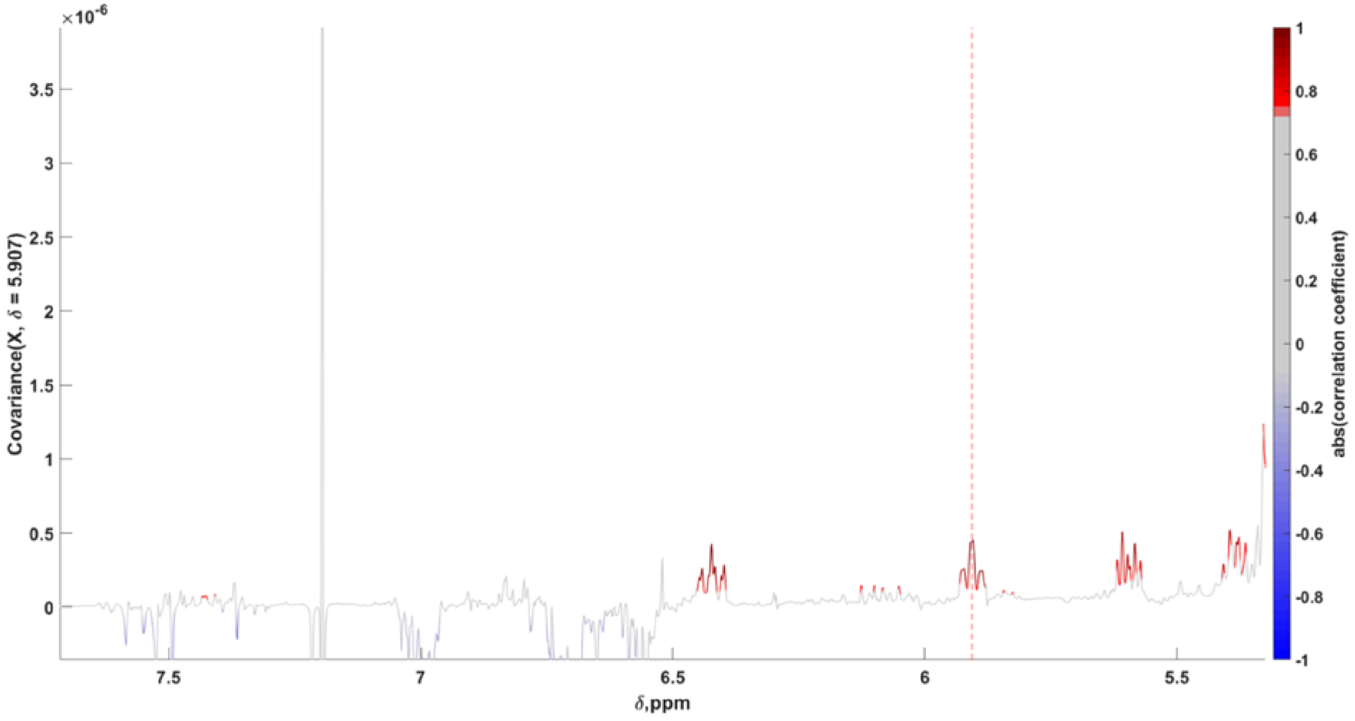

Figure S10. STOCSY 1D pseudo-NMR spectrum of an unknown biomarker. Correlation coefficients to the other signals in the median olive oil NMR spectrum are color-encoded: “driver peak” was at 5.907 ppm.

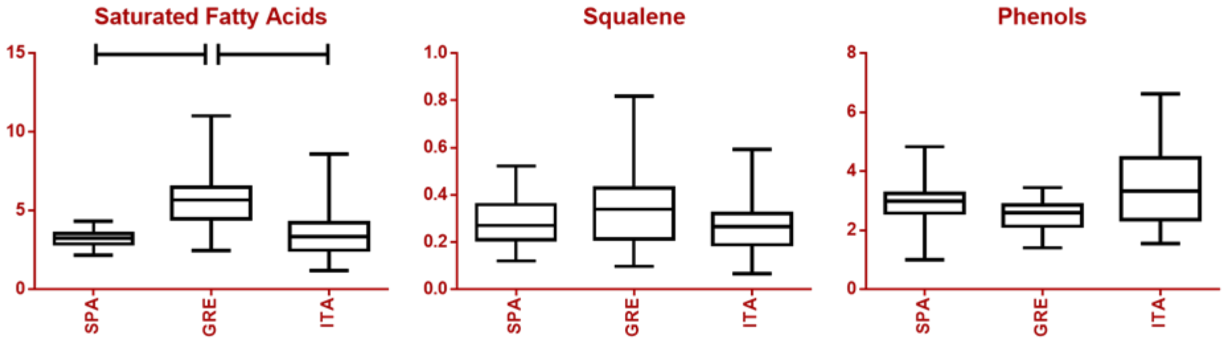

Figure S11. Box plots of a selection of statistically significant markers in the European sample set. Specifically, Saturated fatty acids (SFAs), Squalene and Total Phenols are depicted (vertical axis expressed in normalized intensity). SPA: Spain, GRE: Greece, ITA: Italy.

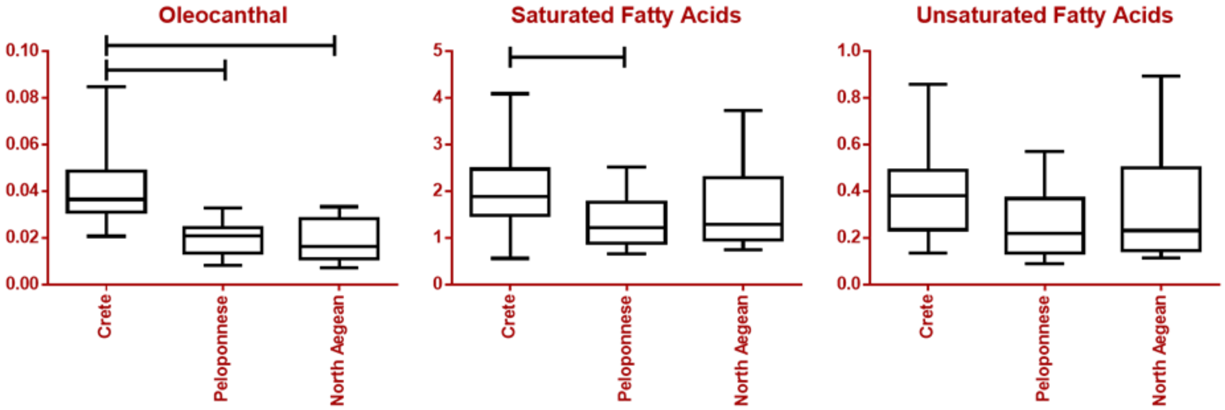

Figure S12. Box plots of a selection of statistically significant markers in the Greek sample set. Specifically, Oleocanthal, SFAs and Unsaturated fatty acids (UFAs) are depicted (vertical axis expressed in normalized intensity).
